# Neuroprotection against beta-amyloid toxicity by the novel estrogen receptor modulator STX requires convergent signaling pathways

**DOI:** 10.3389/fnmol.2025.1670646

**Published:** 2025-09-12

**Authors:** Hun-Joo Lee, Zoe Bostick, John Doherty, Tracy L. Swanson, Martin J. Kelly, Joseph F. Quinn, Nora E. Gray, Philip F. Copenhaver

**Affiliations:** ^1^Department of Cell, Developmental and Cancer Biology, OHSU, Portland, OR, United States; ^2^Department of Psychiatry, OHSU, Portland, OR and VA Portland Health Care System, Portland, OR, United States; ^3^Department of Physiology and Chemical Biology, OHS, Portland, OR, United States; ^4^Department of Neurology, Oregon Health and Science University, Portland, OR, United States; ^5^Parkinson's Disease Research, Education, and Clinical Center, Portland Veterans Affairs Medical Center, Portland, OR, United States

**Keywords:** Alzheimer’s disease, *β*-amyloid, estrogen receptor modulator, SERM, signal transduction, hippocampal neuron, dendrites, mitochondria

## Abstract

**Introduction:**

STX is a synthetic non-steroidal estrogen receptor modulator (SERM) that can provide many of the beneficial effects of 17β-estradiol in the brain without its adverse side effects, via its selective engagement of the membrane estrogen receptor GqMER. Using both neuronal culture assays and transgenic mouse models of Alzheimer’s disease (AD), we have shown that STX protects against the deleterious effects of *β*-amyloid (Aβ), in part by supporting mitochondrial function and synaptic integrity. However, the specific transduction pathways by which STX induces these beneficial responses have not been previously investigated.

**Methods:**

Using the MC65 neuroblastoma model of Aβ toxicity and primary cultures of hippocampal neurons from the 5XFAD mouse model of AD, we analyzed the involvement of different signal transduction pathways associated with STX-dependent responses in other contexts. We used pharmacological methods to test the role of key pathway components in assays of cell viability, neuronal morphology, quantitative immunoblots to analyze pathway engagement, and modulation of the mitochondrial permeability transition pore.

**Results:**

We found that the neuroprotective effects of STX against Aβ toxicity required engagement of the PI3K/Akt/GSK3β pathway. Using well-characterized inhibitors of specific isoforms of the p110 catalytic domain of PI3K, we then showed that this response was predominantly mediated via engagement of the P110δ isoform, with a more modest contribution by P110β. In contrast, targeting the PLC/PKC/PKA pathway (which plays a prominent role in hypothalamic neurons) had a relatively modest effect on the neuroprotective responses induced by STX, while targeting ERK/MAPK signaling had no significant effect.

**Discussion:**

In combination with our previous studies, these results indicate that engagement of GqMER by STX promotes neuroprotective responses via convergent signaling pathways that mitigate the effects of Aβ toxicity on mitochondrial function, synaptic integrity, and neuronal calcium (Ca^2+^) homeostasis. They also provide the framework for testing the mechanisms of STX neuroprotection *in vivo*, using mouse AD models. Since STX has been shown to provide many of the beneficial effects of 17β-estradiol in the brain without its adverse side effects (including feminizing effects in males), these results support the hypothesis that STX might have therapeutic potential in patients at risk of AD.

## Introduction

Because almost two-thirds of patients with Alzheimer’s disease (AD) are postmenopausal women with increased vulnerability to cognitive decline (Altmann et al., 2014; Beam et al., 2018; Nebel et al., 2018; Ferretti et al., 2020; Rahman et al., 2020), hormone replacement with 17β-estradiol (E2) formulations has been considered a promising therapy (Mulnard et al., 2000; Ali et al., 2023). In both animal models and some patients, E2 was found to protect against AD pathogenesis (Zhang G. Q. et al., 2021), but large clinical trials targeting older women revealed serious side effects, including thrombosis, cancer, and increased risk of dementia (Rapp et al., 2003; Shumaker et al., 2004; Manson et al., 2013; Pourhadi et al., 2023). Subsequent trials targeting younger women produced more promising results but still caused many of the same side effects (Song et al., 2020; Nerattini et al., 2023; Pourhadi et al., 2024), while the feminizing effects of E2 precluded its use in men (Abbott et al., 2007; Chen et al., 2020). Selective estrogen receptor modulators (SERMs) targeting specific estrogen receptors (ERs) might provide an improved therapeutic strategy, but SERMs that engage conventional ERs (ERα and ERβ) carry the same risk factors as E2 (Segura-Uribe et al., 2017). By comparison, SERMs that specifically target non-classical estrogen receptors could potentially induce the protective responses of E2 without its side effects. For example, compounds that engage the GPER1 (G protein coupled estrogen receptor 1) protected against Aβ toxicity in mouse models of AD (Kubota et al., 2016; Deng et al., 2017; Wnuk et al., 2020). A caveat is that GPER1 is also expressed in reproductive tissues and in some hormone-sensitive cancers (Olde and Leeb-Lundberg, 2009; Xu et al., 2019), whereby GPER1 agonists carry similar risks as E2 in vulnerable patients. Despite these disappointing results, developing a compound that can provide the neuroprotective benefits of E2 without its adverse side effects remains a promising therapeutic strategy for treating AD (Yao and Brinton, 2012).

As an alternative, the small molecule STX represents a novel non-steroidal compound with these attributes. STX is a synthetic diphenylacrylamide that specifically engages GqMER (Gq-coupled membrane estrogen receptor), independent of ERα, ERβ, and GPER1 (Qiu et al., 2003; Qiu et al., 2006; Kelly and Ronnekleiv, 2015). Although full characterization of this receptor is still in progress, GqMER is expressed by CNS neurons but not by other cell types in the brain or by peripheral reproductive organs (Roepke et al., 2010; Hu et al., 2016). STX is orally bioavailable, readily crosses the blood–brain barrier, and can be safely administered over a wide range of concentrations for months without adverse side effects (Qiu et al., 2003; Qiu et al., 2006; Kelly and Ronnekleiv, 2015). STX treatment also recapitulates the beneficial effects of E2 in models of both menopause and ischemia (Qiu et al., 2006; Roepke et al., 2010; Lebesgue et al., 2010). Equally important, STX does not induce abnormal clotting or oncogenic responses, nor does it cause feminizing effects in males (Qiu et al., 2006), indicating that it might provide a safe and effective treatment for AD in both men and women.

In previous work, we used a variety of cell culture models to show that STX attenuated Aβ neurotoxicity *in vitro*, in part by mitigating Aβ-associated mitochondrial dysfunction and synaptic loss (Gray et al., 2016). In a subsequent study using the 5XFAD mouse model of Aβ pathology (Oakley et al., 2006), we found that oral STX treatment also reduced the levels of reactive astrocytosis and microgliosis surrounding amyloid plaques in the brain, while improving spatial memory (Quinn et al., 2022). Interestingly, the neuroprotective effects of STX were more robust in females than males, consistent with other reports of accelerated pathology in female 5XFAD mice (Forner et al., 2021; Oblak et al., 2021). These results suggest that STX might have therapeutic potential in patients at risk of AD.

Although the mechanisms by which Aβ accumulation in the brain ultimately leads to AD are still under debate, numerous studies have shown that Aβ itself can induce neurodegenerative responses via the misregulation of a variety of signaling pathways, resulting in mitochondrial dysfunction, neuronal Ca^2+^ dyshomeostasis, synaptic dystrophies, and activation of pro-apoptotic pathways that provoke neuronal death (Maccioni et al., 2001; Berridge, 2010; Reitz, 2012). Notably, STX has been shown to modulate several of these signaling pathways in different contexts that might counteract the deleterious actions of Aβ on neuronal health (Roepke et al., 2010; Lin et al., 2009; Inagaki and Etgen, 2013). Accordingly, we have now adapted our cell culture assays of Aβ toxicity to investigate the beneficial mechanisms of STX. Our results indicate that engagement of GqMER by STX promotes neuroprotection via convergent signaling pathways that can mute the deleterious effects of neurotoxic factors linked with AD (including Aβ), mitigating the loss of mitochondrial integrity and synaptic function that impact neuronal health.

## Materials and methods

### Synthesis and preparation of STX

STX was synthesized by Sirius Fine Chemicals GmbH (Bremen, Germany) under contract with the authors of the synthetic protocol for STX, published in Tobias et al. (2006). STX was purified to >95% by HPLC as determined by NMR and LC/MS. Stock solutions of STX (2 mM) were prepared in 100% anhydrous dimethyl sulfoxide (DMSO), which was then diluted to a working concentration of 100 nM in culture medium, as previously described (Gray et al., 2016).

### Animal rearing and use

The following mouse strains were purchased from the Jackson Laboratory (Bar Harbor, ME): C57BL/6 J (strain # 000664, RRID: IMSR_JAX:000664); 5XFAD (strain #034848JAX, RRID: MMRRC_034848-JAX; B6SJLTg(APPSwFlLon, PSEN1*M146L*L286V)6799Vas/Mmjax); and B6SJLF1/J (Strain#:100012, RRID: IMSR_JAX:100012). Male 5XFAD mice were bred with B6SJLF1/J females. Animals were housed and bred in a climate-controlled facility with a 12-h light/12-h dark cycle, with water and diet provided *ab libitum* (Pico Lab Rodent Diet 5LOD; LabDiets, St. Louis, MO). Litters were group-housed (3-4/cage) until pairwise breeding. For all experiments, data collection and analysis was performed by investigators blinded to neuronal genotype and treatment conditions.

### MC65 cell culture

MC65 cells were cultured in 96-well plates in Minimal Essential Media with alpha modifications (MEMα) supplemented with 10% fetal bovine serum (FBS; GIBCO/Life Technologies), 2 mM L-glutamine (Sigma-Aldrich), and 0.1% tetracycline (Sigma-Aldrich). For each experiment, cells were trypsinized and resuspended in Opti-MEM without phenol red (GIBCO/ Life Technologies), then treated with STX or DMSO in the presence and absence of tetracycline (Tet), with or without the following inhibitors (from Selleckchem): LY294002 (5 μM, cat. # S1105), targeting class 1 isoforms of phosphoinositide 3-kinase (PI3K); U73122 (5 μM, cat. # S8011), targeting phospholipase C (PLC); and U0126 (10 μM, cat. # S1102), targeting Mitogen-activated protein kinase 1/2 (MEK1/2). For assays of viability in the presence of inhibitors, cells were plated at 10,000 cells/well in 96 well plates and assessed after 72 h of continuous treatment, compared with Tet-treated cells with or without the addition of STX. MTS (3-(4,5-dimethylthiazol-2-yl)-5-(3-carboxymethoxyphenyl)-2-(4- sulfophenyl)-2H-tetrazolium) assays were performed using the CellTiter 96 Aqueous Non-Radioactive Cell Proliferation method (Promega), as per the manufacturer’s instructions. To investigate the effects of STX on the mitochondrial permeability transition pore (mPTP), replicate MC65 cell cultures were treated at the time of Tet removal with STX or NIM811 (*N*-methyl-4-isoleucine cyclosporin; gift of Drs. Micheal Forte and Justina Šileikytė, OHSU), which inhibits mPTP opening by binding cyclophilin D (a regulator of the mPTP; Waldmeier et al., 2002; Hansson et al., 2004).

### Primary hippocampal neuron cultures

Embryonic 5XFAD mice and their wild type (Wt) littermates were used to generate primary cultures of hippocampal neurons. 5XFAD mice overexpress two transgenes regulated by neural-specific mouse Thy1 promotor elements: human Amyloid Precursor Protein 695 (hAPP) with the Swedish (K670N, M671L), Florida (I716V), and London (V717I) Familial Alzheimer’s Disease (FAD) mutations, plus human presenilin 1 (PS1) with two FAD mutations (M146L and L286V; Oakley et al., 2006). In intact animals, 5XFAD mice begin to accumulate detectable Aβ deposits as early as 2 months, followed by synaptic dysregulation commencing at 4 months, and behavioral deficits followed by neuronal loss that becomes apparent at 6 months (Ohno et al., 2006; Kimura et al., 2010; Oblak et al., 2021; Forner et al., 2021). In primary culture, hippocampal neurons derived from this line exhibit structural and functional changes over 2–4 weeks, including the progressive loss of dendritic complexity and synaptic spine density, compared to neurons from wild type (Wt) control mice (Crowe and Ellis-Davies, 2014; Andersen et al., 2021; Plachez et al., 2023); the misregulation of signal transduction pathways that regulate neuronal viability (Yi et al., 2018; Forest et al., 2021; Medina-Vera et al., 2023); and disrupted Ca^2+^ homeostasis (Skobeleva et al., 2022). For our assays of STX neuroprotection, 5XFAD and Wt littermate embryos were harvested at embryonic day E18 from anesthetized females, following the protocols developed by Kaech and Banker (Kaech and Banker, 2006; Gray et al., 2016). Embryos were genotyped by PCR using DNA extracted from tail samples as previously described (Quinn et al., 2022), and dissected hippocampi from embryos with the same genotype were combined for plating. The hippocampi were minced and trypsinized to generate dispersed neurons, which were quantified with a hemocytometer and plated in enriched Minimal Essential Medium (MEM; GIBCO /Life Technologies) containing 5% FBS (Atlanta Biologicals) and 0.6% glucose (Sigma-Aldrich). After 4 h, the medium was replaced with Neurobasal medium (Gibco 21,103–049) plus 1% 1x GlutaMAX (Gibco/Life Technologies #35050061) and 2% 50x B27 Plus (#A3582801).

### Sholl analysis of dendritic complexity

To determine the optimal concentrations of inhibitors for our assays of dendritic complexity, Wt hippocampal neurons were plated in poly-L-lysine (PLL)-coated 96-well plates at 15,000 neurons per well in enriched MEM. The plates were then immediately placed in an IncuCyte Zoom S3 Live Cell Imaging System, equipped with an environmentally controlled incubator (maintained at 37 °C). At this density, the outgrowth behavior of >10^3^ individual axons per treatment group could be imaged simultaneously. Inhibitors were applied in a range of concentrations based on published *in vitro* assays (6 wells/condition) to identify maximal concentrations that produced only non-significant effects on outgrowth (~10% reduced rate of axonal extension over 100 h). These concentrations were then used to treat sparse neuronal cultures for Sholl analyses of dendritic complexity (Sholl, 1953; Gensel et al., 2010), following our published methods (Gray et al., 2016).

For Sholl analyses, 150,000 hippocampal neurons were plated with enriched MEM in 60 mm dishes, each containing four PLL-coated glass coverslips placed above paraffin wax spacers (Kaech and Banker, 2006; Kaech et al., 2012). After 4 h, the coverslips were flipped into new 60 mm dishes that had been pre-plated with neural stem cell-derived glial cells (provided by Dr. Gary Banker, Jungers Center, OHSU). Coverslips containing hippocampal neurons were then maintained above the glial feeder layers in 6 mL Neurobasal medium. Dishes were fed weekly by replacing 1 mL spent culture medium with 1 mL fresh enriched Neurobasal medium. The first feed (performed at 5 DIV) contained 6 mM cytosine *β*-D-arabinofuranoside hydrochloride (Ara-C; Sigma-Aldrich) to inhibit glial cell proliferation. The first and second feeds (5 DIV and 12 DIV) also contained STX (100 nM) or DMSO in enriched MEM, with or without selective inhibitors or vehicle controls The following inhibitors (from Selleckchem) were used to test the signaling mechanisms of STX, based on the concentrations derived from our titration assays in the IncuCyte Zoom platform: PI-103, specific for P110α/β/*γ*/*δ* (50 nM, cat. # S1038); HS-173, specific for P110α (100 nM, cat. # S7356) 0.1 μM; TGX-221, specific for P110β (10 μM, cat. #S1169); CAL-101, specific for P110δ (1 μM; cat. # S2226); IPI-549 specific for P110γ (10 μM, cat. # S8330); and U73122 (5 μM, cat. # S8011), a pan-Phospholipase C inhibitor (Bleasdale et al., 1990).

At 19 DIV, coverslips were fixed in 4% Paraformaldehyde in PHEM buffer (60 mM PIPES, 25 mM HEPES, 10 mM EGTA, 2 mM MgCl_2_, pH 7.4), and immunostained with Anti-MAP2B (Sigma-Aldrich #M4403, RRID: AB_477193; 3.3 μg/mL) and Goat anti-mouse IgG1-Cy3 (Jackson ImmunoResearch #115–165-205, RRID: AB_2338694; 1.5 μg/mL) to label dendrites, plus DAPI (4′,6-diamidino-2-phenylindole) to label nuclei. Immunostained neurons were imaged with a Zeiss ApoTome.2 on an Axio Imager in the Advanced Light Microscopy Core, OHSU (RRID: SCR_009961). Sholl analyses were performed using ImageJ/Fiji (RRID: SCR_002285; Schindelin et al., 2012) with the plug-in created by Ferreira et al. (2014). At least 90 cells were analyzed in ≥3 independent experiments per treatment condition.

### Evaluation of candidate signaling pathways regulated by STX

For investigating the effects of STX on candidate signaling pathways independent of Aβ, dissociated hippocampal cells from C57BL/6 J embryos were plated in enriched MEM at 150,000 neurons/well in 48-well plates coated with PLL. For some experiments, dissected hippocampi from E18 C57BL/6 mice were purchased from BrainBits (TransnetYX) and used to generate dissociated neurons. Neurons derived by either method exhibited similar responses to STX. After allowing the neurons to attach, plating medium was replaced with 500 μL neurobasal medium, followed by replacement of 200 μL neurobasal medium every 3 days for 1–2 wks. On the day of treatment, the neurons were treated with neurobasal medium containing 100 nM STX or DMSO, with or without selective inhibitors at final working concentrations for 1–60 min. Experiments were terminated by replacing the neurobasal medium with 1,000 μL chilled lysis buffer (1% NP-40, 150 mM NaCl, 50 mM Tris, pH 8) plus PhosSTOP phosphatase inhibitor (Roche 4,906,845,001), protease inhibitor cocktail (Sigma-Aldrich #P8340), and Antipain dihydrochloride (Sigma-Aldrich #A6191). Cells were gently scraped from the coverslips with pipette tips in lysis buffer, and the plates rocked at 4 °C for 1 h. Lysed samples were transferred to Eppendorf tubes, centrifuged at 12,000 rpm for 10 min, and quantified by NanoDrop with BCA protein assay kits (Pierce/Thermo Fisher Scientific). Samples were then immediately analyzed by western blotting protocols or aliquoted and frozen at −80 °C for later use. Selective inhibitors targeting specific signal transduction pathways were initially titrated by treating replicate wells of neurons with a range of concentrations, which were then analyzed in Western blots with antibodies against the targeted phosphorylated proteins (summarized below). For these short-term culture experiments, inhibitors were tested at the following concentrations: PI-103, 1–500 nM; HS-173,1–500 μM; TGX-221, 0.2–200 μM; CAL-101, 0.1–100 μM; and IPI-549, 0.2–200 μM. Their effects on STX-dependent phosphorylation responses were then analyzed using optimized concentrations that induced only non-significant effects on the targeted pathway (as summarized above).

### Neuronal assays with synthetic β-amyloid peptide oligomers

Oligomeric forms of human Aβ_1-42_ were prepared following the methods developed by Stine et al. (2003) and Stine et al. (2011). Briefly, 1 mg lyophilized human Aβ_1-42_ (Echelon Biosciences #641–15) was dissolved in hexafluoro-2-propanol to create a 1 mM solution of Aβ monomers. The stock solution was transferred to 0.5 mL Eppendorf tubes (22 μL/tube), air-dried overnight, speed-vac dried at room temperature (RT) for 1 h, and stored at -20 °C. One day before an experiment, individual aliquots were resuspended in 5 μL anhydrous DMSO to create a stock concentration of 221 μM, bath sonicated for 10 min, diluted into 95 mL chilled DMEM/F-12 (without phenol red; Thermo-Fisher # 21041025), vortexed briefly, and incubated overnight at 4 °C. The following day, samples were centrifuged for 10 min at 12 K rpm at 4 °C, and then added to sparse cultures of hippocampal neurons with or without STX (as described above) at a final concentration 1–10 μM. Based on published protocols (Pham et al., 2010; Dahlgren et al., 2002; Pryor et al., 2012), the extent of oligomerization was monitored in western blots of Tris-Tricine polyacrylamide gels labeled with anti-human Aβ_1-16_ (6E10; BioLegend #80304, RRID: AB_271585). The effects of Aβ_1-42_ on markers of neuronal Ca^2+^ dysregulation were monitored by immunohistochemistry, using a rabbit anybody (Cell Signaling Technology #2614, RRID: AB_2168458; 1:500) against Calcineurin A (CaN), the catalytic subunit of Calcineurin, or a rabbit anybody (Santa Cruz Biotechnology #sc-13036, RRID: AB_650208; 1:200) against Nuclear Factor of Activated T cells cytoplasmic 4 (NFATc4). Primary antibodies were detected with goat-anti-rabbit Alexa-Fluor 488-coupled secondary antibodies (Thermo-Fisher #A-11008, RRID: AB_143165; 1:1,000) and counterstained with DAPI. To quantify changes in the subcellular distribution of CaN and NFATc4 as markers of Ca^2+^ overload (Saraf et al., 2018; Chen et al., 2022), ratios of the relative fluorescent intensities in the nucleus (overlapping with DAPI) versus the cytoplasm were quantified in Fiji, following the methods of Hudry et al. (2012).

### Western blotting

Cell lysates were denatured for 5 min at 100 °C. After brief centrifugation, the samples (2 μg total protein per lane) were separated by electrophoresis in 26-well Criterion XT 12% Bis-Tris polyacrylamide gels (BioRad #3450119). Each gel included samples from all treatment groups (STX vs. vehicle, +/− inhibitors) to allow for quantitative comparisons of relative protein levels, analyzed by an investigator blinded to treatment conditions. Electrophoresed samples were then transferred to low-fluorescence PVDF membranes (Immun-Blot; BioRad #162–0263) using a semi-dry transfer apparatus in Tris/Glycine buffer. Gels were periodically stained with GelCode Blue (Thermo-Fisher # 24590) to monitor transfer efficiency, and the membranes were rinsed repeatedly in blocking buffer TBST plus 1% Blotto nonfat dry milk; Rockland #B501-0500; 1% blot-qualified Bovine Serum Albumin (BSA; Promega W3841); and 50 mM NaF. Membranes were subsequently incubated for 1 h at RT with primary antibodies (summarized below), diluted in 1X Tris-buffered saline plus 0.1% Tween 20 (TBST). The membranes were then rinsed in 1X TBST for 5 min with gentle rocking and incubated with appropriate secondary antibodies for 30 min at RT. The following primary antibodies from Cell Signaling Technology were used in this analysis (antibodies made in rabbit; used at 1:1500): anti-phospho-Akt (Ser473, CST #4060, RRID: AB_2315049); anti-Pan-Akt (CST #4691; RRID: AB_915783); anti-phospho-GSK3β (Ser9, CST #5558; RRID: AB_10013750); anti-Pan GSK3β (CST #12456, RRID: AB_2636978); anti-phospho-p44/42 MAPK (ERK1/2); Thr202/Tyr204, CST #4370, RRID: AB_2315112); anti-Pan-p44/42 MAPK (ERK1/2; CST #4695, RRID: AB_390779). Primary antibodies were detected with HRP-conjugated donkey-anti-rabbit IgG from Jackson ImmunoResearch (#711–035-152, RRID: AB_10015282) at 1:10,000. After final rinsing in 1X TBST for 5 min, the membranes were incubated with enhanced chemiluminescence substrate (ECL; SuperSignal West Pico PLUS; Thermo-Fisher #34579) and visualized sequentially on both X-ray film and on a c600 Azure imager. The digital Azure images were subsequently analyzed using AzureSpot software and analyzed in Excel. For sequential analyses of phosphorylated versus non-phosphorylated proteins, membranes were first labeled with phospho-specific antibodies, then stripped in BlotFresh Western Blot Stripping Reagent Ver. II (SignaGen Laboratories # SL100324), monitored for residual chemiluminescent signal, and then labeled with non-phospho-specific (pan) antibodies in TBST plus 5% Blotto nonfat dry milk. Lastly, the membranes were stripped again and labeled with mouse-anti-GAPDH as a loading control (R&D Systems # 5718, RRID: AB_2278695; 0.5 μg/mL), detected with HRP-conjugated donkey-anti-mouse IgG (Jackson ImmunoResearch; #715–035-150, RRID: AB_2340770, diluted to 1:10,000. To analyze protein expression levels, background subtraction was first performed for each band per sample using the ‘rolling ball’ function in AzureSpot software, and relative values were determined as ratios to GAPDH intensities for each band. To compare samples across multiple immunoblots, intensity values were normalized against the average values obtained from replicate lysate samples of control (vehicle-treated) neurons. Quantification was performed blind to genotype and treatment conditions.

### Statistical analysis

Statistical differences among treatment groups were analyzed using one- or two-way ANOVA with Tukey’s post-hoc tests for multiple comparisons in Prism 10 (RRID: SCR_002798). Histograms showing means +/− SD or +/− SEM were used to display the data graphically (as summarized in each figure legend), overlaid with dots indicating individual values (omitted in the complex histograms showing Sholl analyses, for visual clarity). Effects were considered significant at *p* < 0.05. Statistically significant differences are indicated in each figure via the following convention: *p < 0.05, **p < 0.01, ***p < 0.001 ****p < 0.0001.

## Results

Experiments in different model systems have revealed that STX can regulate neuronal responses via a variety of signal transduction pathways, depending on the cell type and context. In different classes of hypothalamic neurons, stimulation of GqMER by STX led to the sequential activation of phospholipase C (PLC), protein kinase Cδ (PKCδ) and protein kinase A (PKA) (Qiu et al., 2006; Qiu et al., 2008; Kelly and Ronnekleiv, 2008; Kelly and Ronnekleiv, 2009; Smith et al. O' Neill, 2013; Nag and Mokha, 2014; Hu et al., 2016; Conde et al., 2016). By comparison, the neuroprotective effects of STX in murine models of ischemia required the rapid activation of the PI3K/Akt (Protein Kinase B) signaling cascade (Lebesgue et al., 2010; Etgen et al., 2011), while the anti-nociceptive response regulated by STX in noradrenergic spinal neurons specifically required.

Extracellular Signal-Regulated Kinase (ERK/MAPK) signaling (Nag and Mokha, 2014). In many contexts, the effects of STX involve convergent regulation of several of these pathways, reflecting the complex responses elicited by E2 that are often recapitulated by GqMER activation (Qiu et al., 2006; Lin et al., 2009; Malyala et al., 2008; Kelly and Qiu, 2010). Accordingly, we adapted our cell culture assays to investigate the signaling mechanisms by which STX protects against Aβ toxicity.

**Figure 1:**
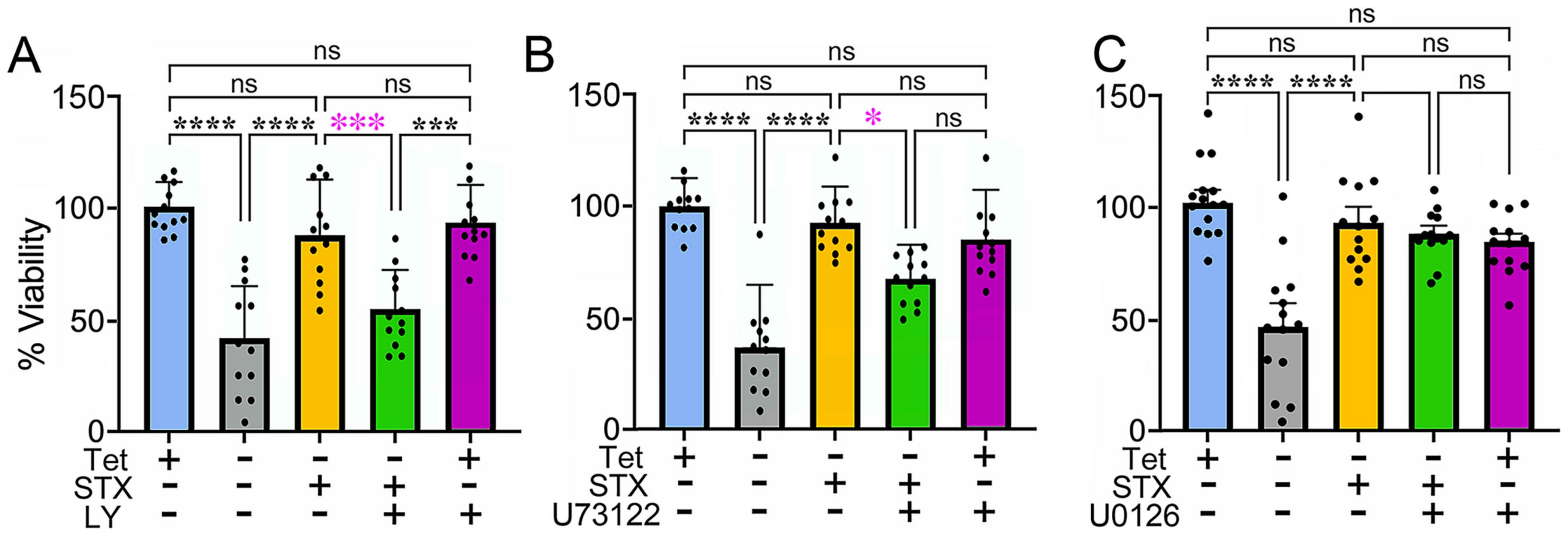
Analysis of candidate signaling pathways required for the protective effects of STX in MC65 cells. Histograms indicate cell viability in different treatment conditions, normalized to the Tet^+^ (control) group. **(A)** Compared to control MC65 cell cultures maintained in the presence of Tet (Tet^+^; blue histogram), induction of A*β* production by Tet removal (Tet^−^) resulted in extensive cell death by 72 h (gray histogram), while treatment with 100 nM STX prevented the loss of viability caused by Aβ (yellow histogram). Treatment with the pan-PI3K inhibitor LY294002 (5 μM, green histogram) significantly reduced the protective effects of STX against Aβ toxicity in Tet^−^ cultures (indicated by magenta asterisks), whereas this concentration of LY294002 had no detectable effect in Tet + control cultures (purple histogram). Representative images of MC65 cell cultures in the different treatment conditions are shown in Supplementary Figure 1A. **(B)** Using the same treatment protocol, the broad-spectrum PLC inhibitor U73122 (5 μM; green histogram) caused a partial reduction in the protective effect of STX (indicated by magenta asterisk). **(C)** the MEK1/2 inhibitor U0126 (10 μM; green histogram) had no detectable effect (indicated by the magenta ‘ns’). N = 12 separate sets of 96 well cultures (grown in triplicate) per treatment condition. Histograms show means +/− S. D. overlaid with individual values (dots). Statistics: one-way ANOVA with Tukey’s post-hoc comparisons; alpha = 0.05. In **A**, *F* = 23.19, R^2^ = 0.6278. In **B**, *F* = 18.72, R^2^ = 0.5765. In **C**, *F* = 14.16, R^2^ = 0.5073. *p < 0.05; ***p < 0.001; ****p < 0.0001; ns = not significant.

In previous experiments, we used the MC65 neuroblastoma model to show that STX protects against Aβ-induced cell death *in vitro* (Gray et al., 2016). MC65 cells conditionally express the C99 C-terminal fragment of Amyloid Precursor Protein (APP) under the control of a Tet-responsive promoter: upon Tet removal (Tet^−^), C99 is rapidly expressed and then cleaved by endogenous secretases to generate cytotoxic Aβ peptides (predominantly Aβ_1-40_ and Aβ_1-42_), resulting in progressive cell death (Sopher et al., 1996; Maezawa et al., 2006; Woltjer et al., 2007). As an initial test of the protective effects of STX in this assay, we treated replicate MC65 cultures with broad-spectrum inhibitors targeting each candidate pathway in the presence or absence of Tet (to provoke Aβ-induced cell death). Consistent with our past work, stimulating Aβ production resulted in extensive cell death by 72 h (Figures 1A–C, gray histograms), whereas treatment with STX largely prevented this toxic response (yellow histograms). Notably, co-treatment with the pan-PI3K inhibitor LY294002 blocked the protective effect of STX (Figure 1A, green histogram; *p* < 0.001) at a concentration (5 μM) that did not significantly increase cell death in control (Tet^+^) cultures (purple histogram). Representative images of MC65 cells in each treatment condition are shown in Supplementary Figure 1. By comparison, co-treatment with the broad-spectrum PLC inhibitor U73122 (Figure 1B) resulted in a smaller reduction in STX-induced protection (*p* < 0.05), while co-treatment with the broad-spectrum MEK1/2 inhibitor U0126 (Figure 1C) did not have a significant effect, again using concentrations that did not affect cell viability in control cultures. These results suggest that engagement of GqMER by STX protects cells against Aβ toxicity predominantly via engagement of the PI3K pathway with a lesser contribution associated with PLC-dependent signaling, but it does not require ERK/MAPK signaling.

**Figure 2:**
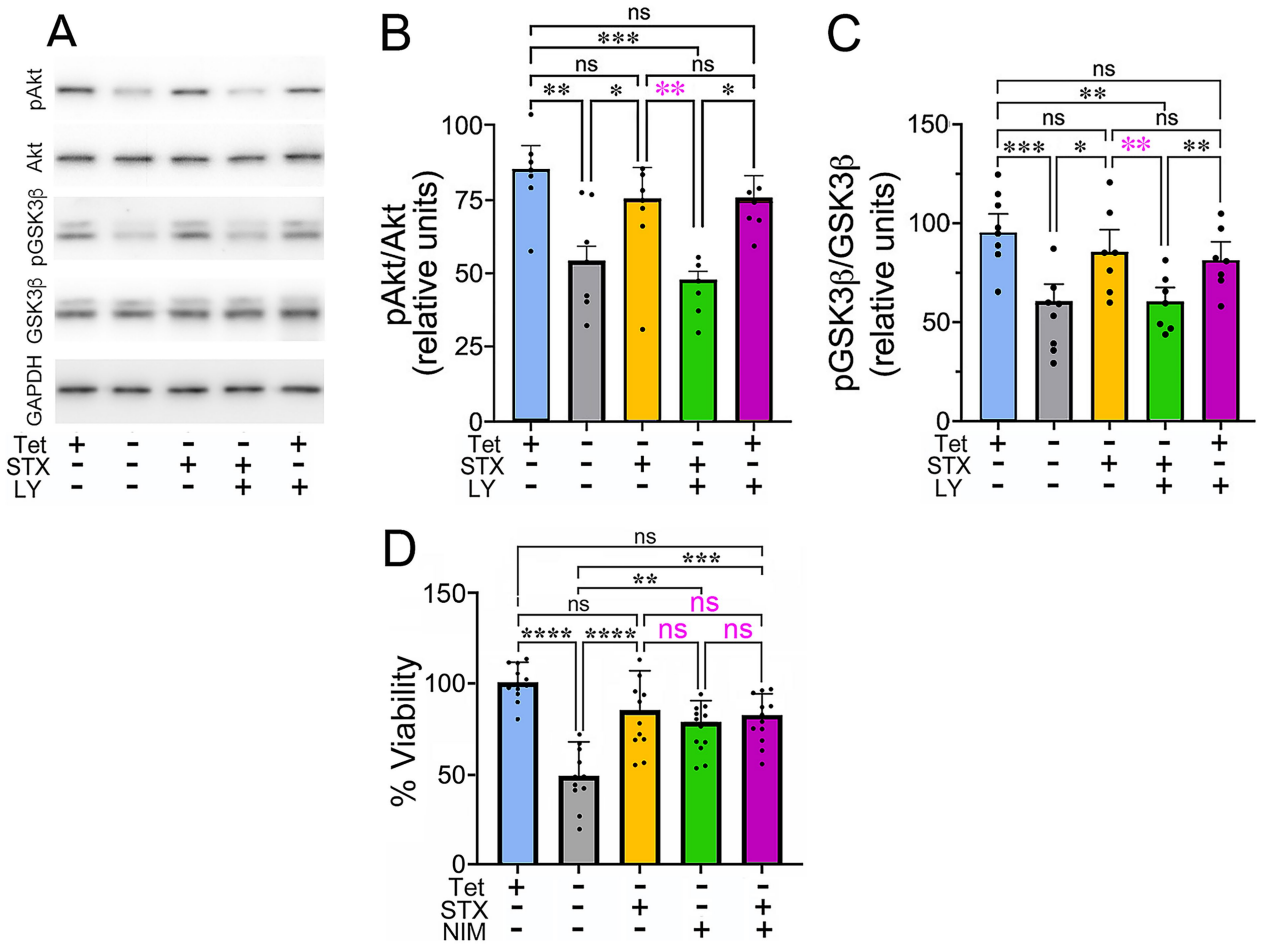
STX protects against the decline in Akt and GSK3β phosphorylation caused by Aβ in MC65 cells. **(A)** Western blot of lysates from MC65 cultures grown in the presence or absence of Tet (to induce Aβ production), with or without STX and the pan-PI3K inhibitor LY294002 (LY). In Tet^−^ conditions (to induce Aβ production), pAkt and pGSK3β levels were significantly reduced, compare to Tet^+^ cultures, while pan-Akt and pan-GSK3β levels were unchanged. STX treatment maintained pAkt and pGSK3β at levels similar to that of Tet^+^ control cultures. In contrast, LY294002 inhibited the protective effect of STX at a concentration (5 μM) that did not significantly alter pAkt and pGSK3β levels in Tet^+^ cultures (in the absence of Aβ). For this analysis, the same western blots were immunostained sequentially with antibodies against pAkt, pGSK3β, pan-Akt, pan-GSK3β, and GAPDH. Full-sized immunoblots are shown in Supplementary Figure 2. **(B)** Quantification of pAkt/pan-Akt levels in MC65 cell cultures, normalized to GAPDH (shown as relative units). Compared to control cultures (Tet^+^; blue histogram), removal of Tet to induce Aβ production (Tet^−^; gray histogram) resulted in a significant reduction in pAkt levels, whereas pan-Akt levels were not significantly altered. Treatment with STX (yellow histogram) protected against the decline in pAkt caused by Aβ, while co-treatment with the pan-PI3K inhibitor LY294002 blocked the protective effect of STX (green histogram). This concentration of LY294002 did not significantly alter pAkt levels in control (Tet^+^) cultures (purple histogram). **(C)** Quantification of pGSK3β/pan-pGSK3β levels, normalized to GAPDH (shown as relative units); histogram colors indicate equivalent treatment conditions shown in **A**. In the presence of Aβ (Tet^−^), pGSK3β levels were significantly reduced compared to control (Tet^+^) cultures, while pan-GSK3β levels were not significantly affected. STX treatment prevented the decline in pGSK3β caused by Aβ, an effect that was blocked by LY294002. **(D)** Treatment with STX (yellow histogram) protected against the effects of Aβ on the viability of MC65 cells (Tet^−^; gray histogram), similar to Figure 1. Treatment with the CypD inhibitor NIM811 (to prevent chronic mPTP opening) produced a similar protective effect on cell viability (green histogram), while treatment with NIM811 + STX did not increase viability more than NIM811 alone (magenta histogram). Histograms indicate cell viability in different treatment conditions, normalized to the Tet^+^ (control) group. N ≥ 10 separate sets of 96 well cultures (grown in triplicate) per treatment condition. Statistics: one-way ANOVA with Tukey’s post-hoc comparisons; Histograms show means +/− S. D. overlaid with individual values (dots). Statistics: in **B**, alpha = 0.05, *F* = 7.942, R^2^ = 0.4138. in **C**, alpha = 0.05, *F* = 8.262, R^2^ = 0.4234. In **D**, alpha = 0.05, *F* = 14.51, R^2^ = 0.5134. *p < 0.05; **p < 0.01; ***p < 0.001; ****p < 0.0001; ns = not significant.

An important downstream target of PI3K is glycogen synthase kinase 3 beta (GSK3β), which when chronically activated provokes the loss of mitochondrial function and neuronal Ca^2+^ homeostasis, as well as increased APP cleavage and Aβ production, hyperphosphorylation of tau, and neuronal dystrophy (Crews and Masliah, 2010; Lauretti et al., 2020; Zhao et al., 2024). Conversely, stimulation of PI3K induces the phosphorylation and activation of Akt, which in turn phosphorylates and inactivates GSK3β, restricting these deleterious responses (Endo et al., 2006; Salcedo-Tello et al., 2011; Hernandez et al., 2013). Similarly, engagement of the PLC/PKC pathway also can phosphorylate GSK3β to restrict its activity in a variety of contexts (Shin et al., 2002; Moore et al. O' Neill, 2013; Tang et al., 2011). In our MC65 cell assays, we found that induction of Aβ production following Tet removal resulted in a significant decline in basal levels of phosphorylated Akt (pAkt) and phosphorylated GSK3β (pGSK3β), corresponding to enhanced GSK3β activity (Figures 2A–C), whereas STX treatment (yellow histograms) maintained pAkt (Figure 2B) and pGSK3β (Figure 4C) at levels similar to control (Tet+) cultures (blue histograms). In contrast, co-treatment with the pan-PI3K inhibitor LY294002 (green histograms) blocked the effects of STX on Akt and GSK3β phosphorylation levels, consistent with its effects in our MC65 cell death assay (Figure 1). Uncropped immunoblots from this experiment are shown in Supplementary Figure 2.

**Figure 3:**
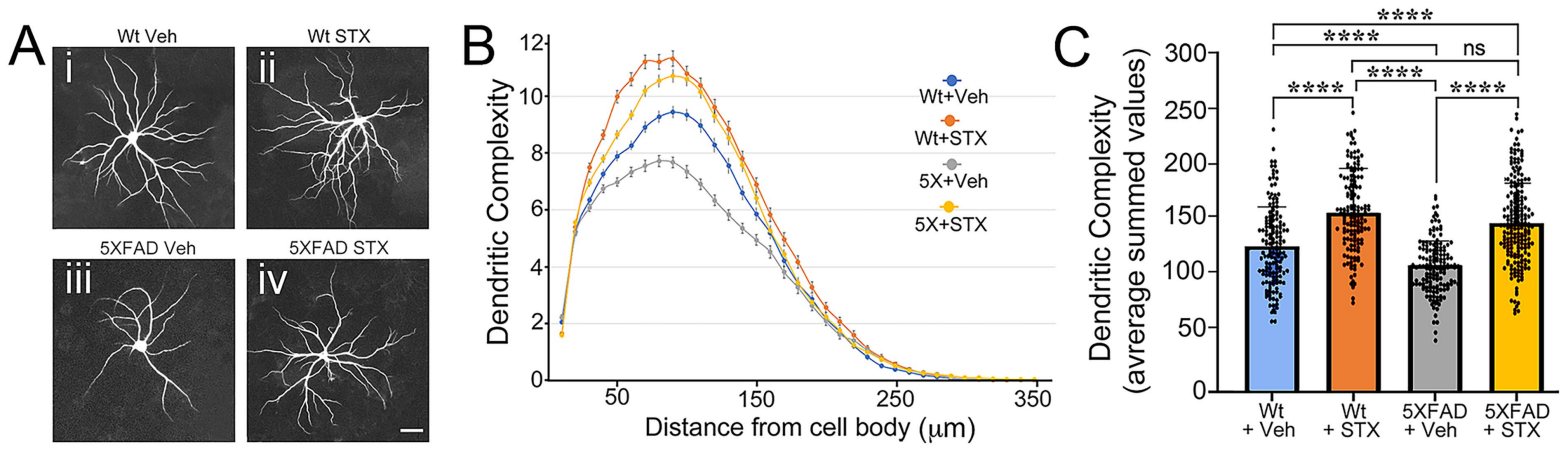
STX protects against Αβ toxicity in primary cultures of 5XFAD hippocampal neurons. **(A)** Examples of mouse E18 hippocampal neurons from 5XFAD mice or wild type (Wt) littermate controls that were grown as sparse cultures for 14 days above glial feeder layers, treated with STX or vehicle (0.01% DMSO in enriched MEM) for 7 days, and then immunostained with anti-MAP2 antibodies to label dendrites. *Panel i*: Wt neuron with vehicle. *Panel ii*: Wt neuron treated with STX. *Panel iii*: 5XFAD neuron treated with vehicle. *Panel iv*: 5XFAD neuron treated with STX. Scale = 20 μm. **(B)** Sholl analyses of dendritic complexity in Wt versus 5XFAD neurons (measured with respect to distance from the cell body). Compared to vehicle-treated Wt neurons, dendritic complexity was significantly reduced in vehicle-treated 5XFAD neurons. Treatment with STX protected against the loss of dendritic complexity in 5XFAD neurons and slightly improved complexity in Wt neurons. **(C)** Quantification of Sholl analyses shown in **B**, represented as averaged summed values of dendritic crossings at distances of 10–300 μm from the cell body. Compared to vehicle-treated Wt neurons (blue histogram), vehicle-treated 5XFAD neurons (gray histogram) exhibited a significant loss of dendritic complexity by 3 weeks. STX treatment protected against the loss of dendritic complexity in 5XFAD neurons (yellow histogram) and significantly improved complexity in Wt neurons (orange histogram). N ≥ 150 neurons per group. Histograms show means +/− S. D. overlaid with individual values. Statistics: one-way ANOVA with Tukey’s post-hoc comparisons; alpha = 0.05, *F* = 57.70, R^2^ = 0.2095. ****p < 0.0001; ns = not significant.

**Figure 4:**
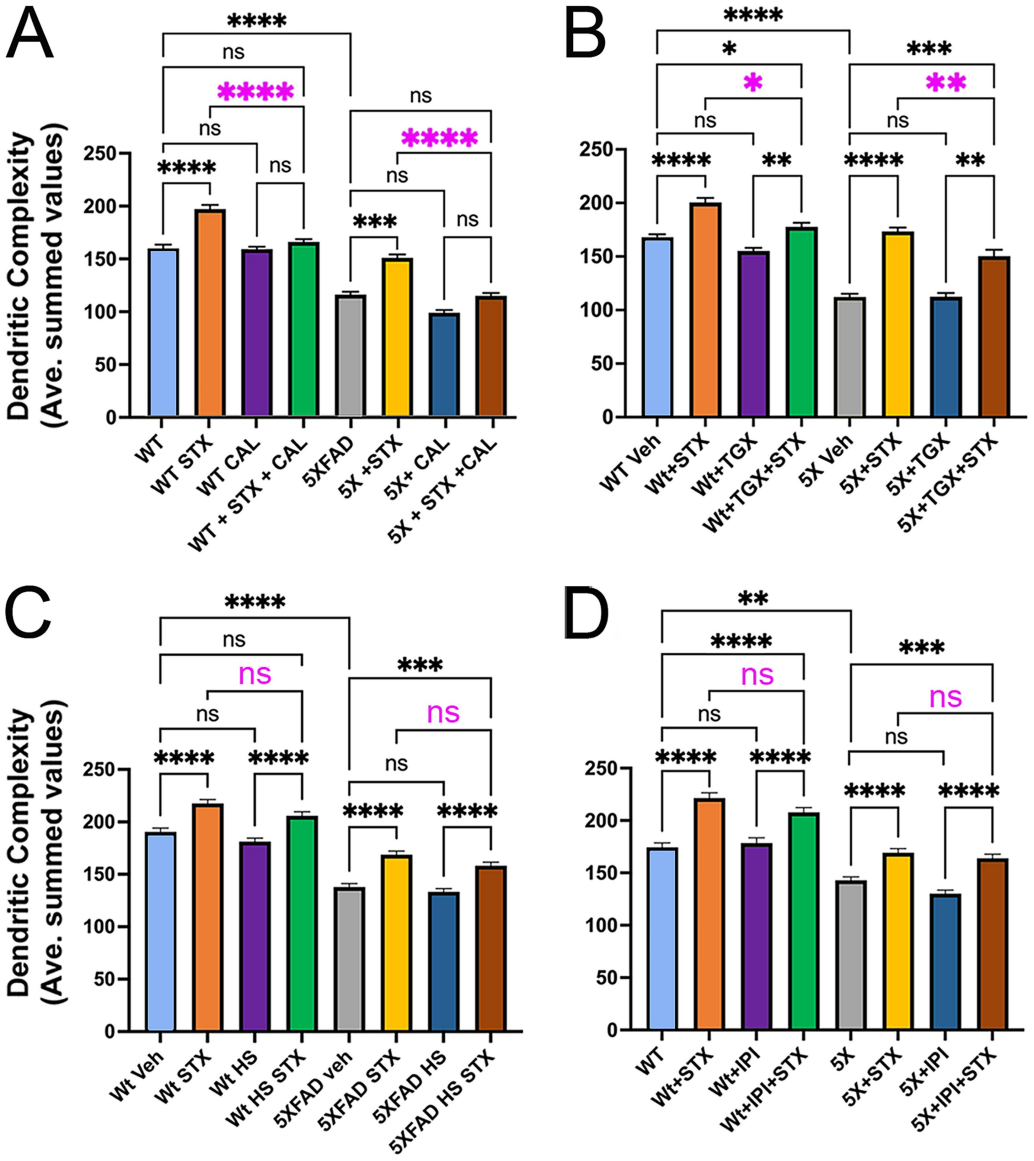
The protective effects of STX require specific isoforms of the p110 catalytic subunits of PI3K. Graphs derived from Sholl analyses of dendritic complexity in replicate cultures of hippocampal neurons from Wt and 5XFAD mice, represented as averaged summed values of dendritic crossings at distances of 10–300 μm from the cell body. **(A)** Compared to DMSO-treated Wt neurons (light blue histogram), STX-treated Wt neurons (yellow histogram) exhibited a significant increase in dendritic complexity. Treatment with p110*δ*-specific inhibitor CAL-101 (1 μM; green histogram) significantly reduced the beneficial effect of STX on Wt neurons (highlighted by magenta asterisks) but had no detectable effect on basal dendritic complexity at this concentration (purple histogram). Similarly, STX induced a significant increase in dendritic complexity in 5XFAD neurons (yellow histogram) compared to DMSO-treated 5XFAD neurons (gray histogram). Treatment with 1 mM CAL-101 had no detectable effect on basal levels of dendritic complexity in 5XFAD neurons (dark blue histogram) but significantly reduced the beneficial effect of STX (brown histogram; highlighted by magenta asterisks). N ≥ 107 neurons per group. Histograms show means +/− SEM. Representative images of hippocampal neurons grown as sparse cultures above glial feeder layers for the different treatment conditions in this experiment are shown in Supplementary Figure 1B. Panels **B–D** show the same labeling convention as **A. (B)** Treatment with p110β-specific inhibitor TGX-221 (TGX; 10 μM) caused a less significant reduction in the beneficial effects of STX in both Wt neurons (green histogram) and 5XFAD neurons (brown histogram); at this concentration, TGX-221 had no significant effect on either Wt neurons (purple histogram) or 5XFAD neurons (dark blue histogram). N ≥ 175 neurons per group. **(C)** Treatment with p110α-specific inhibitor HS-173 (HS; 100 nM) caused no significant reduction in the beneficial effects of STX on Wt neurons (green histogram) or 5XFAD neurons (brown histogram). N = 150 neurons per group. **(D)** Treatment with p110*γ*-specific inhibitor IPI-149 (IPI; 10 μM) also caused no significant reduction in the beneficial effects of STX on Wt neurons (green histogram) or 5XFAD neurons (brown histogram). N = 120 neurons per group. Treatment concentrations were selected based on an initial analysis of neurite outgrowth using the Zoom IncuCyte platform (described in Methods). Statistics: one-way ANOVA with Tukey’s post-hoc comparisons; alpha = 0.05. In **A**, *F* = 73.56, R^2^ = 0.2808. in **B**, *F* = 70.25, R^2^ = 0.2563. in **C**, *F* = 1.909, R^2^ = 0.1522. in **D**, *F* = 1.299, R^2^ = 0.2471. *p < 0.05; **p < 0.01; ***p < 0.001; ****p < 0.0001; ns = not significant.

In many diseases, chronic GSK3β activation induces irreversible opening of the mitochondrial permeability transition pore (mPTP), resulting in mitochondrial swelling, loss of ATP production, Ca^2+^ dysregulation, and cell death (Rao et al., 2014; Bernardi et al., 2015; Perez et al., 2018; Gray et al., 2016). Conversely, compounds that stimulate PI3K-Akt activity can restrict the deleterious effect of GSK3*β* activation on mitochondrial function (Rasola et al., 2010; Maurer et al., 2014; Yang et al., 2017; Wu et al., 2022), suggesting that STX might also protect neurons via engagement of this pathway. To test whether the phosphorylation and inactivation of GSK3β by a candidate compound protects against chronic mPTP activation, a common strategy is to compare its effects with cyclosporine A (CsA), an inhibitor of cyclophilin D (CypD), which regulates the mPTP (Zulian et al., 2014; Warne et al., 2016). However, since CsA also affects a number of other intracellular pathways (Liddicoat and Lavelle, 2019), we instead compared the effects of STX with *N*-methyl-4-isoleucine cyclosporin (NIM811), a more selective inhibitor of CypD that robustly inhibits mPTP opening (Waldmeier et al., 2002; Hansson et al., 2004; Zulian et al., 2014). In MC65 cells, both STX and NIM811 protected against Aβ-induced cytotoxicity to a similar degree (Figure 2D, yellow and green histograms), whereas combined treatment with NIM811 + STX did not further improve viability (magenta histogram). These results are consistent with the hypothesis that engagement of the PI3K/Akt pathway by STX mitigates chronic mPTP opening by GSK3β, thereby supporting mitochondrial function. Whether this response is specifically regulated via the phosphorylation and inactivation of GSK3β in this assay remains to be determined.

Based on the foregoing experiments, we adapted our protocols for testing the beneficial effects of STX in neuronal culture assays of Aβ toxicity. In previous work, we showed that STX protected isolated hippocampal neurons from the Tg2576 mouse model of AD, mitigating the effects of Aβ on mitochondrial function and synaptic complexity (Gray et al., 2016). For the current study, we used neurons harvested from the more aggressive 5XFAD model, which provided an efficient assay for analyzing the signaling pathways required for the neuroprotective effects of STX *in vitro*. When grown as sparse cultures positioned above a supportive glial feeder layer (Kaech and Banker, 2006), 5XFAD neurons exhibited a gradual decline in dendritic branching after 3 weeks in culture compared to neurons from Wt littermate control mice, while treatment with STX increased the extent of branching in neurons from both genotypes (Figure 3A). Using Sholl analyses of dendritic complexity (Sholl, 1953; Gensel et al., 2010), we observed a significant loss of dendritic branching in 5XFAD neurons by three weeks in culture (compared to control neurons), whereas STX treatment prevented this decline and enhanced dendritic branching in Wt cultures (Figures 3B,C). These results are consistent with our previous studies using neurons from Tg2576 mice (Gray et al., 2016), demonstrating that STX has a robust neuroprotective effect in different models of A*β* pathology.

As noted above, our studies using MC65 cells suggest that engagement of the PI3K/Akt/GSK3β pathway might be required for STX to protect against Aβ toxicity. Using a similar protocol, we found that the pan-PI3K inhibitor LY 294002 significantly reduced the protective effects of STX in 5XFAD neurons (not shown). However, since broad-spectrum compounds of this type are likely to have off-target effects (Gharbi et al., 2007), we used a suite of validated inhibitors targeting the catalytic subunits of individual PI3K isoforms (p110α, β, *γ*, *δ*) for this analysis. Whereas all four isoforms are expressed by neurons, they are associated with distinct functions in the CNS (Jia et al., 2008; Koren and Bentires-Alj, 2013; Choi et al., 2014). In particular, P110β can regulate autophagic responses during neurodegeneration (Dou et al., 2010) and contributes to STX-dependent responses in hypothalamic neurons (Smith et al., 2013; O' Neill, 2013), while P110δ has been shown to regulate axonal regeneration (Eickholt et al., 2007) and may indirectly affect Aβ accumulation (Martinez-Marmol et al., 2019). In addition, P110α has been shown to promote dendritic growth in some contexts (Sanchez-Castillo et al., 2022) and might ameliorate cognitive decline in AD models, while P110γ plays a role in certain types of synaptic plasticity and potentially modulates excitotoxic responses in the hippocampus (Kim et al., 2011; Gross and Bassell, 2014).

In preparation for this analysis, we first tested each inhibitor over a range of concentrations using the Zoom IncuCyte platform, which identified maximal concentrations that produced non-significant effects on neuronal outgrowth (~10% reduced rate of axonal extension over 100 h). These concentrations were then used to treat sparse neuronal cultures for Sholl analyses of dendritic complexity as in index of neuronal health. Using this method, we found that 1.0 μM CAL101 (a specific inhibitor of p110δ; Lannutti et al., 2011; Low et al., 2014) significantly reduced the beneficial effects of STX in both Wt and 5XFAD neurons (Figure 4A, magenta asterisks) without causing deleterious responses in neurons of either genotype at this concentration. By comparison, treatment with 10 μM TGX-221 (a specific inhibitor of p110β; Straub et al., 2006; Chaussade et al., 2007) had a less significant effect on STX-induced protection (Figure 4B, magenta asterisks), while 100 nM HS-173 (specific for p110α; Lee et al., 2013) and 10 μM IPI-549 (specific for p110γ; Evans et al., 2016) did not affect STX-dependent responses in this assay (Figures 4C,D). These results suggest that the neuroprotective effects of STX are predominantly mediated via engagement of the P110δ isoform of PI3K, with a more modest contribution by P110β.

**Figure 5:**
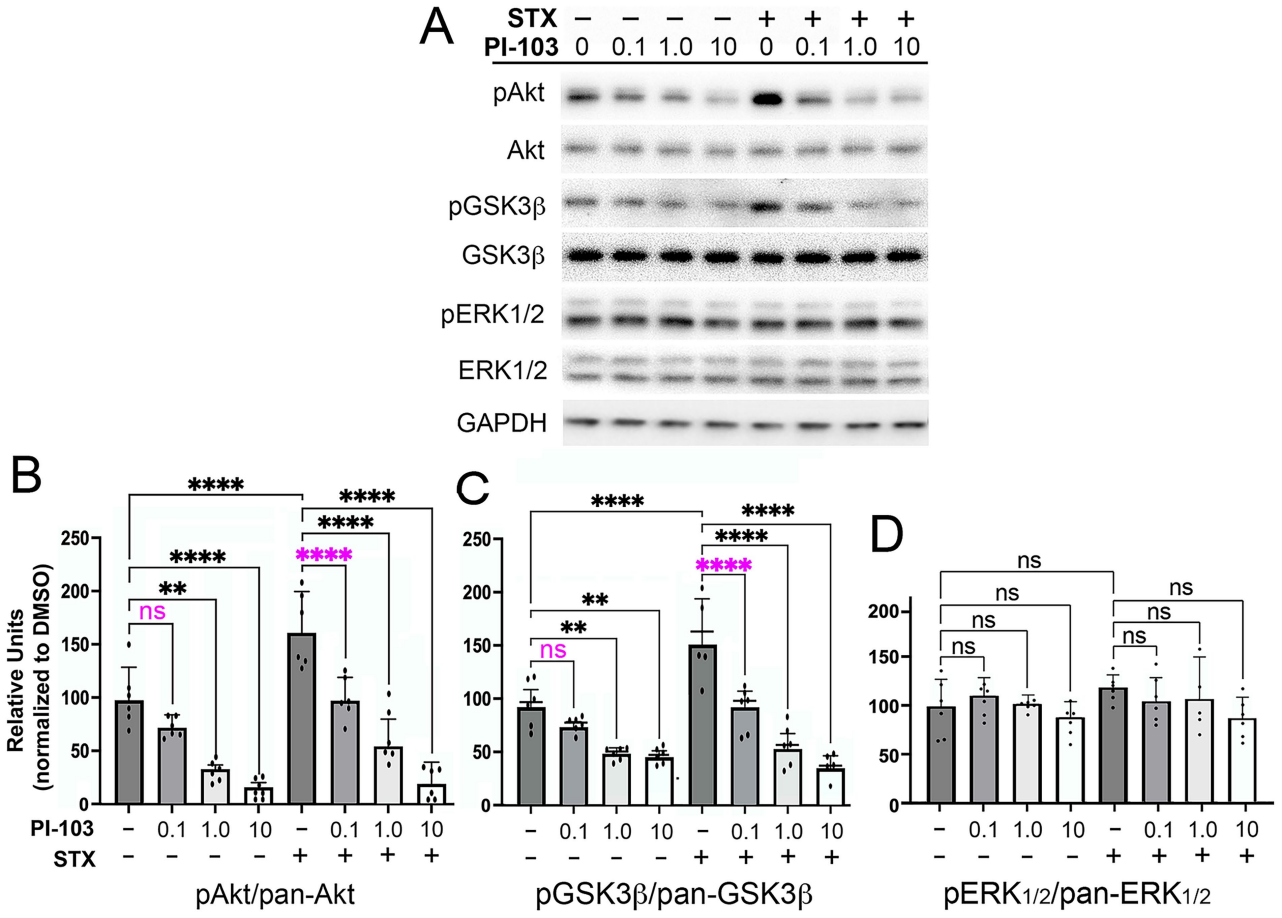
The PI3K inhibitor PI-103 blocked the phosphorylation of Akt and GSK3β induced by STX in hippocampal neurons. **(A)** Example of a western blot of lysates prepared from hippocampal neuron cultures grown in dense cultures that were treated with control medium (with 0.01% DMSO) or STX (100 nM), with or without PI-103 (targeting p110α/β/γ/δ). Representative images of hippocampal neurons grown in dense cultures for the different treatment groups in this experiment are shown in Supplementary Figure 1C. For this analysis, the same western blot was immunostained sequentially with antibodies against pAkt, pan-Akt, pGSK3β, pan-GSK3β, and GAPDH. Full-sized immunoblots are shown in Supplementary Figure 3. Treatment with STX induced a significant increase in pAkt and pGSK3β levels without affecting pan-Akt or pan-GSK3β levels and had no detectable effect on pERK1/2 or pan-ERK1/2 levels. **(B)** Quantification of the effects of different PI-103 concentrations on STX-induced phosphorylation responses. At 0.1 μM, PI-103 inhibited the effects of STX on pAkt levels (magenta asterisks) but did not significantly reduce basal levels in DMSO-treated neurons. Higher PI-103 concentrations reduced pAkt levels in both DMSO-treated and STX-treated neurons. **(C)** At 0.1 μM, PI-103 inhibited the effects of STX on pGSK3β levels (magenta asterisks) but did not significantly reduce basal levels in DMSO-treated neurons. **(D)** Treatment with PI-103 over a range of concentrations did not affect pERK1/2 levels in either DMSO-treated or STX-treated neurons. In this experiment, STX alone induced only a slight increase in pERK1/2 levels above control levels. Treatment concentrations were selected based on an initial analysis of neurite outgrowth using the Zoom IncuCyte platform (described in Methods). Histograms show means +/− S.D. overlaid with individual values. Statistics: N = 6; one-way ANOVA with Tukey’s post-hoc comparisons; alpha = 0.05. In **B**, *F* = 32.64, R^2^ = 08510. In **C**, *F* = 28.76, R^2^ = 0.8342. In **D**, *F* = 1.168, R^2^ = 0.1697. **p < 0.01; ****p < 0.0001; ns = not significant.

To further investigate the role of different PI3K isoforms in transducing the beneficial effects of STX, we used high-density cultures of hippocampal neurons (60 K per well in 48-well plates) to analyze the pathway by which STX regulates the phosphorylation of Akt and GSK3β. As an initial test, we treated neurons with STX plus the PI3K inhibitor PI-103 (targeting p110α/β/γ/δ; Westhoff et al., 2009; Bagci-Onder et al., 2011) over a range of concentrations for 5 min, and then quantified pAkt, pGSK3β, and pERK1/2 levels in western blots of the cell lysates (normalized to their non-phosphorylated isoforms). As shown in Figures 5A–C, PI-103 induced a concentration-dependent reduction in both pAkt and pGSK3β levels, while STX treatment induced a significant increase in both phosphoproteins compared to control cultures. Notably, the response to STX was inhibited by PI-103 at a concentration (0.1 μM) that did not significantly reduce basal levels of pAkt and pGSK3b in cultures lacking STX (Figures 5B,C; magenta asterisks), whereas pERK1/2 levels were unaffected by STX or PI-103 (Figure 5D). Uncropped immunoblots are shown in Supplementary Figure 3. These results are consistent with the effects of the pan-PI3K inhibitor LY 294002 on STX-induced phosphorylation responses in MC65 cells (Figure 2).

**Figure 6:**
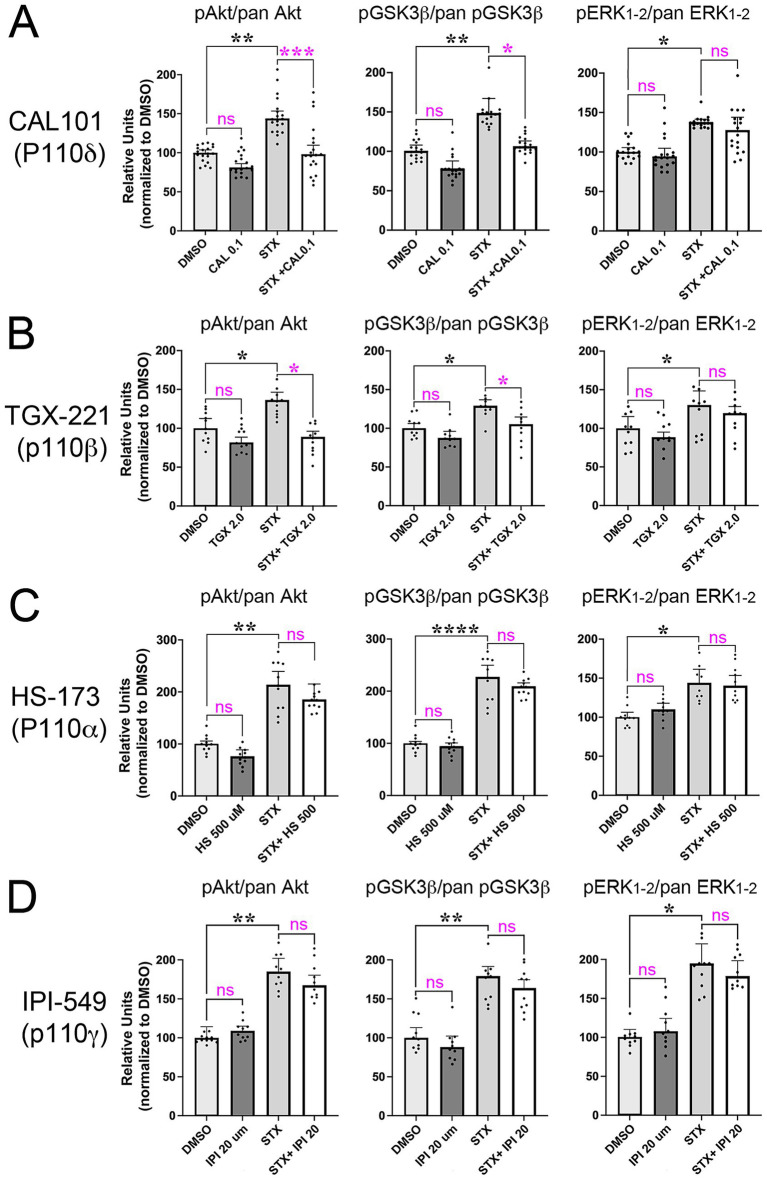
STX-induced phosphorylation of Akt and GSK3β in hippocampal neurons requires specific p110 catalytic subunits of PI3K. Maximal concentrations of inhibitors targeting specific p110 isoforms that did not significantly affect basal pAkt and pGSK3β levels were identified as in Figure 5. **(A)** In cultured Wt hippocampal neurons, STX induced a significant increase in pAkt, pGSK3β, and pErk1/2 levels (normalized to pan Akt, GSK3β, and ERK1/2, respectively). Treatment with the p110δ-specific inhibitor CAL-101 (0.1 μM) blocked the increase in pAkt and pGSK3β phosphorylation induced by STX without affecting basal phosphorylation levels in DMSO-treated neurons. **(B)** Treatment with the p110β-specific inhibitor TGX-221 (2 μM) caused a less significant inhibition of STX-induced phosphorylation of Akt and GSK3β. **(C,D)** Treatment with the p110α-specific inhibitor HS-173 (500 μM); **(C)** or the p110γ-specific inhibitor IPI-549 (20 μM); **(D)** had no significant effect. Full-sized immunoblots for each treatment group are shown in Supplementary Figures 4–7. Histograms show means +/− S.E.M. overlaid with individual values Statistics: N = 10; one-way ANOVA with Tukey’s post-hoc comparisons; alpha = 0.05. In **A**: for pAkt/pan Akt: *F* = 11.09, R^2^ = 0.3160; for pGSK3β/pan GSK3β: *F* = 7.787, R^2^ = 0.2557; for pERK1/2/pan ERK1/2: *F* = 4.326, R^2^ = 0.163. In **B**: for pAkt/pan Akt: *F* = 7.570, R^2^ = 0.3622; for pGSK3β/pan GSK3β: *F* = 7.918, R^2^ = 0.3975; for pERK1/2/pan ERK1/2: *F* = 6.314, R^2^ = 0.3448. In **C**: for pAkt/pan Akt: *F* = 23.51, R^2^ = 0.6621; for pGSK3β/pan GSK3β: *F* = 32.34, R^2^ = 0.781; for pERK1/2/pan ERK1/2: *F* = 4.115, R^2^ = 0.2553. In **D**: for pAkt/pan Akt: *F* = 6.575, R^2^ = 0.3813; for pGSK3β/pan GSK3β: *F* = 9.375, R^2^ = 0.4386; for pERK1/2/pan ERK1/2: *F* = 2.624, R^2^ = 0.1794. *p < 0.05; **p < 0.01; ****p < 0.0001; ns = not significant.

Using this protocol, we identified concentrations of each isoform-specific PI3K inhibitor that did not induce significant effects on basal phosphoprotein levels in short-term assays, and then tested their ability to inhibit the stimulation of Akt and GSK3β phosphorylation by STX. The results of this analysis are summarized in Figure 6. The p110δ-specific inhibitor CAL-101 (0.1 μM) blocked the increase in pAkt and pGSKβ phosphorylation induced by STX, relative to their non-phosphorylated isoforms (Figure 6A). By comparison, the p110β-specific inhibitor TGX-221 had a smaller inhibitory effect on the phosphorylation responses induced by STX (Figure 6B), whereas the p110α inhibitor HS-173 and the p110γ inhibitor IPI-549 had no effect (Figures 6C,D). Uncropped immunoblots used for analyzing the effects of each inhibitor are shown in Supplementary Figures 4–7. These experiments are consistent with the results of our Sholl-based assays using long-term neuronal cultures (Figure 4), indicating that STX promotes neuroprotective responses in hippocampal neurons via the PI3K/Akt/GSK3β signaling pathway, predominantly via engagement of P110δ (and to lesser degree via P110β). Of note is that STX treatment also induced a detectable increase in pERK1/2 levels in these short-term assays, matching past reports (Lin et al., 2009; Nag and Mokha, 2014; Inagaki and Etgen, 2013). However, treatment with the p110-specific inhibitors had no effect on this response (as expected), while our experiments using MC65 cells indicated that ERK1/2 activation does not significantly contribute to the protective effects of STX against Aβ toxicity (Figure 1).

**Figure 7:**
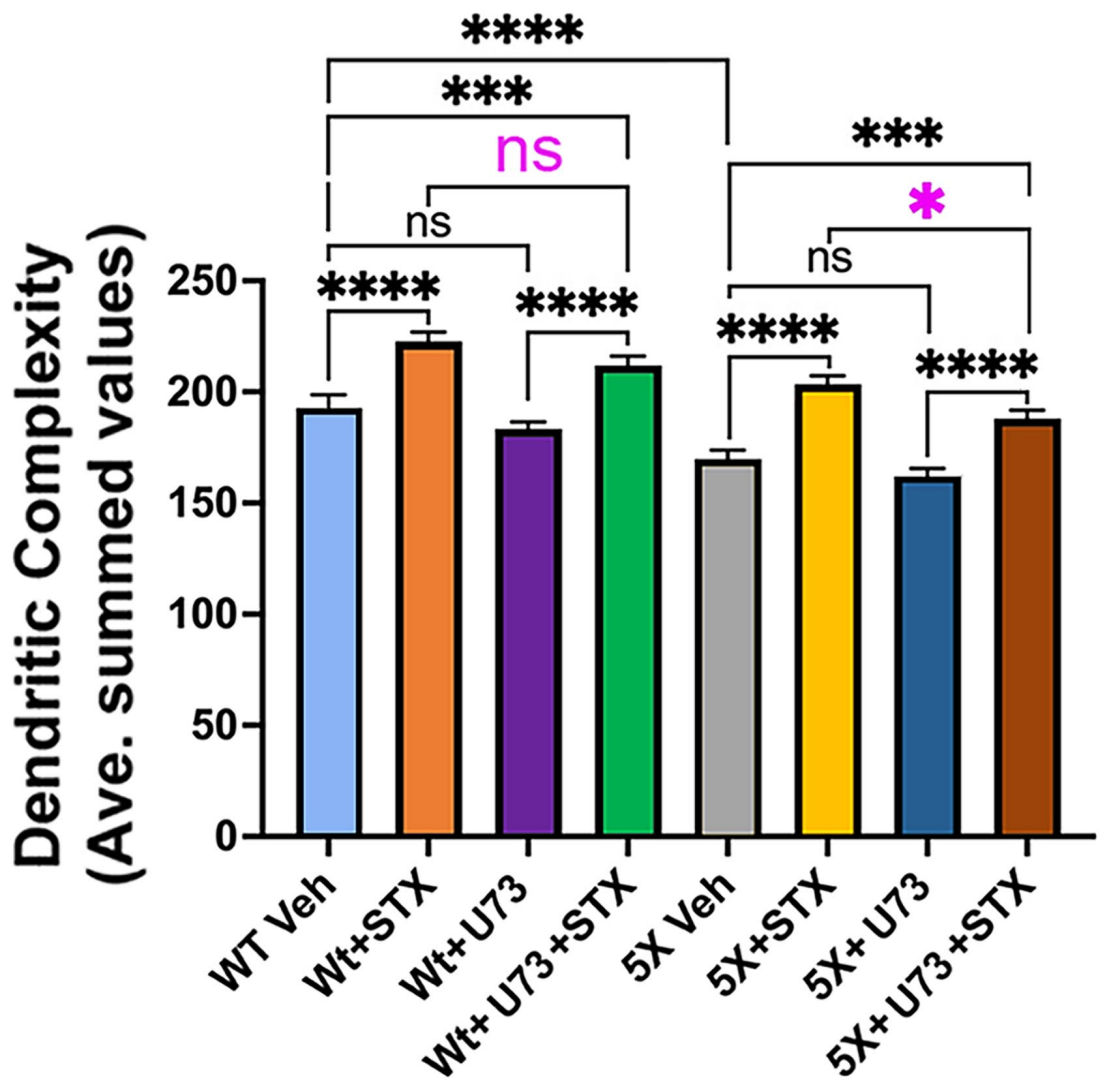
The PLC inhibitor U73122 also partially reduced the beneficial effects of STX on dendritic complexity in cultured hippocampal neurons. Graph derived from Sholl analyses of dendritic complexity in replicate cultures of hippocampal neurons from Wt and 5XFAD mice, represented as averaged summed values of dendritic crossings at distances of 10–300 μm from the cell body. Compared to DMSO-treated Wt neurons (light blue histogram), STX-treated Wt neurons (yellow histogram) exhibited a significant increase in dendritic complexity, similar to Figure 3. Treatment with PLC inhibitor U73122 (5 μM; green histogram) caused a slight reduction in the beneficial effect of STX on Wt neurons (not significant) and had no detectable effect on basal dendritic complexity (purple histogram). STX also induced a significant increase in dendritic complexity in 5XFAD neurons (yellow histogram) compared to DMSO-treated 5XFAD neurons (gray histogram). U73122 did not affect dendritic complexity in 5XFAD neurons at this concentration (dark blue histogram) but caused a significant reduction in the beneficial effect of STX (brown histogram; highlighted by magenta asterisk). N ≥ 90 neurons per group. Histograms show means +/− SEM. Treatment concentrations were selected based on an initial analysis of neurite outgrowth using the Zoom IncuCyte platform (described in Methods). Statistics: one-way ANOVA with Tukey’s post-hoc comparisons; alpha = 0.05. *F* = 44.28, R^2^ = 0.2702. *p < 0.05; ***p < 0.001; ****p < 0.0001; ns = not significant.

Because engagement of GqMER can also promote neuronal responses via engagement of the PLC/PKCδ/PKA pathway (most notably in hypothalamic neurons; Qiu et al., 2006; Qiu et al., 2008; Kelly and Ronnekleiv, 2008; Kelly and Ronnekleiv, 2009; Smith et al., 2013; Hu et al., 2016; Conde et al., 2016), we also used Sholl assays to investigate whether treatment with the pan-PLC inhibitor U73122 also impacted the beneficial effects of STX in hippocampal neurons. Interestingly, we found that inhibiting PLC with 5 μM U73122 also partially reduced the ability of STX to protect against the loss of dendritic complexity in 5XFAD neurons (*p* < 0.05), similar in magnitude to the effects of blocking p110β (Figure 7, magenta asterisk), although it did not significantly alter the response to STX in Wt neurons. Of note is that engagement of the PLC/PKC pathway can also phosphorylate GSK3β to restrict its activity in a variety of contexts (Shin et al., 2002; Moore et al., 2013; Tang et al., 2011). These results suggest that the coordinated engagement of PI3K/Akt and PLC/PKCδ signaling by STX promotes neuroprotective responses that mitigate the effects of Aβ toxicity, thereby supporting mitochondrial function, synaptic integrity, and neuronal viability (Gray et al., 2016; Quinn et al., 2022).

## Discussion

Despite promising studies showing that estrogen replacement in animal models protected against cognitive deficits (Hara et al., 2015; Lejri et al., 2018), clinical trials using E2 in older patient groups resulted in unacceptable adverse outcomes, including some instances of increased dementia (Maki et al., 2019; Savolainen-Peltonen et al., 2019). Likewise, SERMs that target conventional ERs carry many of the same risk factors as E2 and therefore are not acceptable for treating AD patients (Segura-Uribe et al., 2017; Coman et al., 2017). In contrast, our studies on STX (which selectively engages GqMER but not conventional ERs) suggest that it can provide the neuroprotective benefits of E2 signaling without its adverse side effects, potentially representing a viable therapeutic strategy for treating AD (Yao and Brinton, 2012; Wnuk et al., 2020; Cipriano et al., 2024). Experiments using both rodent and primate models of menopause showed that peripherally administered STX readily crossed the blood–brain barrier and restored homeostatic control of body temperature without inducing the side effects of E2, including feminizing effects in males (Qiu et al., 2003; Qiu et al., 2006; Roepke et al., 2010; Kaufman et al., 2013). Likewise, STX prevented the loss of hippocampal neurons in a rat model of ischemia, recapitulating the neuroprotective effects of E2 (Lebesgue et al., 2010; Inagaki and Etgen, 2013). Using neuroblastoma models and cultured hippocampal neurons, we subsequently demonstrated that STX protected against Aβ-associated mitochondrial dysfunction and synaptic loss *in vitro* (Gray et al., 2016). More recently, we showed that treating 5XFAD mice *in vivo* with oral STX attenuated markers of Aβ-associated mitochondrial and synaptic toxicity in the brain, while reducing reactive gliosis (Quinn et al., 2022). Oral STX also protected against the decline in hippocampal-dependent memory in these experiments, supporting the hypothesis that it might have therapeutic potential for treating AD.

For the current study, we adapted our cell culture protocols to investigate the signal transduction pathways required for the protective effects of STX. As its name indicates, GqMER is a membrane-associated receptor coupled to the heterotrimeric G protein Gαq that regulates a variety of intracellular signaling pathways normally activated by E2, independent of subsequent transcriptional responses (Qiu et al., 2003; Roepke et al., 2009; Vail and Roepke, 2019). Not surprisingly, the signaling pathways induced by STX are cell type-specific, reflecting the diverse functions of E2 in the nervous system. Extensive work by the Kelly lab has demonstrated prominent roles for the PLC/PKC/PKA pathway in transducing STX-dependent responses in different types of hypothalamic neurons (Smith et al., 2014; Kelly and Ronnekleiv, 2015). For example, in NPY/AgRP neurons, engagement of this pathway by STX resulted in enhanced GABAergic activation of inward-rectifying potassium channels, thereby *decreasing* their excitability (Smith et al., 2013). Conversely, in POMC neurons, STX *increased* membrane excitability via PLC/PKCδ/PKA signaling by inhibiting coupling between GABA_B_ receptors and potassium channels (Qiu et al., 2008; Vail and Roepke, 2019). Similarly, STX induces a rapid PLC-dependent excitatory response in GNRH neurons, resulting in enhanced Ca^2+^ oscillations and GnRH release (Kenealy et al., 2011). These results augment many other studies showing that activation of Gαq-coupled membrane receptors frequently regulate cellular responses via PLCβ activation, which in turn promotes diacylglycerol (DAG) and inositol 1,4,5-trisphosphate (IP3)-dependent regulation of a wide variety of effector pathways (Falkenburger et al., 2013; Dickson et al., 2013).

However, activation of Gq-coupled receptors like GqMER can also regulate a variety of other transduction pathways in different contexts (Mizuno and Itoh, 2009; Kamato et al., 2017; Arang et al., 2023), including both the PI3K/Akt and ERK/MAPK pathways (Della Rocca et al., 1997; Ballou et al., 2006; Murga et al., 1998; Leroy et al., 2007; New et al., 2007; Desale et al., 2021). For example, in animal models of brain ischemia, STX recapitulates the neuroprotective effects of E2 via engagement of the PI3K/Akt pathway, resulting in the phosphorylation and inactivation of GSK3β and other Akt targets associated with neurodegenerative responses (Lebesgue et al., 2010; Etgen et al., 2011; Inagaki et al., 2012). Alternatively, the anti-nociceptive response regulated by STX in noradrenergic spinal neurons specifically requires ERK/MAPK signaling (Nag and Mokha, 2014). Notably, several studies have shown that STX-induced responses require convergent signaling by a combination of these pathways: in arcuate nucleus neurons, STX attenuates inhibitory responses via a combination of PLC/PKC/PKA, PI3K, and nNOS signaling (Conde et al., 2016), while in GnRH neurons, STX engages both the PI3K/Akt and PLC/PKC pathways (Kelly and Qiu, 2010), and the effects of STX in cell culture models of ER signaling involved both the PI3K/Akt and ERK/MAPK pathways (Lin et al., 2009).

It was therefore notable that targeting PLC activation with broad-spectrum inhibiters in both MC65 cells (Figure 2B) and hippocampal neurons (Figure 7) caused only a partial reduction in the protective effects of STX, indicating that other signaling mechanisms are required for this response. In contrast, we found that pan-PI3K inhibitors robustly inhibited STX-dependent responses in both assays (Figures 2A, 5), whereas targeting ERK/MAPK signaling had no significant effect (Figure 1C). Moreover, our experiments using inhibitors targeting specific P110 isoforms of PI3K showed that the protective effects of STX on dendritic complexity were predominantly mediated by the P110δ isoform, with an additional contribution by P110β (Figure 5). Likewise, the PI3K-dependent effects of STX on the phosphorylation of Akt and GSK3β were most strongly inhibited by CAL-101 (targeting p110δ), with a lesser effect by TGX-221 (targeting p110β) and no effect by inhibitors targeting p110α and p110γ (Figure 6).

Whereas the activation of PLC (particularly different PLCβ isoforms) by receptor-coupled Gαq has been extensively documented (Wu et al., 1992; Hubbard and Hepler, 2006; Litosch, 2016), the mechanisms by which Gαq regulates the PI3K/Akt pathway are less well understood. In transfected cell lines, Gαq was shown to *inhibit* PI3K/Akt signaling by directly binding to p110α but not p110γ (Ballou et al., 2003; Ballou et al., 2006). In contrast, Gαq was found to *stimulate* PI3K signaling in adipocytes via p110α and p110γ activation (Imamura et al., 1999), and Gαq was required for bradykinin-dependent PI3K activation in transfected HeLa cells (Xie et al., 2000). In some contexts, Gβγ as well as Gαq can regulate the activity of different PI3K isoforms (including p110β and p110γ; Brock et al., 2003; Dbouk et al., 2012; Desale et al., 2021), while separate stable pools of Gαq-coupled receptor complexes may regulate distinct signaling responses within a cell, depending on their engagement by different ligands (Golebiewska and Scarlata, 2008). Whether stimulation of GqMER by STX induces Gαq-dependent regulation of p110δ directly or indirectly remains to be determined.

It should be noted that the PI3K/Akt pathway has been linked to the neuroprotective effects of a variety of candidate therapeutic compounds in AD models (Long et al., 2021; Desale et al., 2021; Pan et al., 2024), including SERMs that engage the non-canonical estrogen receptor GPER1 (Deng et al., 2017; Roque and Baltazar, 2019; Upadhayay et al., 2023). However, other reports have shown that chronic overactivation of this pathway can also have deleterious effects on neurons (Chiang et al., 2010; Perluigi et al., 2014; O'Neill et al., 2012), highlighting the need for therapeutic approaches that can normalize PI3K/Akt signaling (and regulate GSK3β-dependent responses) at physiological levels (O' Neill, 2013; Razani et al., 2021). In this regard, we found that STX protected against the decline in pPI3K and pAkt levels caused by Aβ in both MC65 cells (Figure 2) and cultured hippocampal neurons (Figure 6), while inducing only incremental increases in their phosphorylation in control cultures. These results are consistent with past work showing that treating intact mice with STX produced neuroprotective responses without causing adverse neurological responses that are associated with the chronic misregulation of this pathway (Qiu et al., 2006; Lebesgue et al., 2010; Roepke et al., 2010; Quinn et al., 2022).

An emerging theme in many neurodegenerative diseases is that chronic GSK3β activation provokes the irreversible opening of mitochondrial permeability transition pore (mPTP), resulting in mitochondrial rupture, cytochrome C release, loss of ATP production, disrupted cellular Ca^2+^ homeostasis, and neuronal death (Perez et al., 2018; Kalani et al., 2018; Kent et al., 2021; Baev et al., 2024; Yang, 2025). Hence, therapeutic strategies that regulate the phosphorylation and inactivation of GSK3β without impacting other aspects of neuronal homeostasis might provide an important therapeutic strategy for preventing chronic mPTP activation, thereby protecting the brain against neurotoxic factors (Yang et al., 2017; Arrazola et al., 2017; Sun et al., 2019; Rippin and Eldar-Finkelman, 2021). In this regard, both the PI3K/Akt and PLC/PKC pathways engaged by STX can promote the phosphorylation and inactivation of GSK3β (Fang et al., 2002; Tang et al., 2011; Zhou et al., 2013; Jaworski et al., 2019), consistent with our data showing that STX induced a rapid increase in GSK3β phosphorylation in both MC65 neuroblastoma cells and hippocampal neurons (Figures 2, 6). Likewise, our evidence suggesting that STX protects against the effects of Aβ on chronic mPTP activation (similar to the CypD inhibitor NIM811; Figure 2D) supports our previous studies showing that STX is mitoprotective both *in vitro* and *in vivo* (Gray et al., 2016; Quinn et al., 2022).

Mitochondrial dysregulation also impairs Ca^2+^ buffering in neurons (Cabezas-Opazo et al., 2015; Cai and Tammineni, 2017), impacting synaptic function and neuronal integrity. Concurrently, prolonged Ca^2+^ dyshomeostasis provokes the activation and nuclear translocation of the Ca^2+^-dependent phosphatase calcineurin (CaN), which in turn stimulates the activation and nuclear translocation of the transcription factor NFATc4 (the most abundant NFAT isoform in neurons; Ho et al., 1994; Mackiewicz et al., 2023). Of note is that prolonged CaN-NFAT activation induces the same pattern of dystrophic neurites and dendritic spine loss as Aβ both *in vitro* and *in vivo* (Wu et al., 2010) and has been used as a marker for chronic depolarization linked with cognitive decline in AD (Abdul et al., 2009; Hopp et al., 2018; Lara Aparicio et al., 2022). In an initial study, we found that acute treatment of mouse hippocampal neurons with synthetic Aβ_42_ oligomers caused a significant increase in nuclear levels of CaN and NFATc4, whereas STX mitigated these responses (Supplementary Figures 8A,B). Although other factors linked with neurodegeneration can also provoke CaN and NFAT activation (Cardoso and Oliveira, 2005; Reese and Taglialatela, 2011; Norris, 2018), our results suggest that engagement of convergent signaling pathways by STX protects against the loss of neuronal Ca^2+^ homeostasis, which is a hallmark feature of AD pathology (Berridge, 2010; Swerdlow et al., 2014; Wang et al., 2025). Based on past work showing that STX recapitulates the beneficial effects of E2 in regulating intracellular Ca^2+^ dynamics in hypothalamic neurons (Kenealy et al., 2011), we are currently using brain slice preparations from 5XFAD mice to investigate the signaling mechanisms by which STX helps maintain Ca^2+^-dependent aspects of normal hippocampal synaptic function.

Our results also support previous studies showing that STX protects against the loss of mitochondrial gene expression and ATP production caused by Aβ (Gray et al., 2016; Quinn et al., 2022), thereby mitigating key factors that provoke elevated production of reactive oxygen species and oxidative stress linked with neuronal disfunction in AD (Tonnies and Trushina, 2017; Ionescu-Tucker and Cotman, 2021; Plascencia-Villa and Perry, 2023). Likewise, our findings are consistent with the model that engagement of GqMER-dependent signaling by STX can attenuate both the apoptotic (Krishtal et al., 2017; Kumari et al., 2023) and necroptotic (Liu et al., 2015; Balusu and De Strooper, 2024) responses associated with Aβ toxicity, based on the reduction in cell death observed in both MC65 cells (Figure 1) and SH-SY5Y cells treated with Aβ (Gray et al., 2016). Whether engagement of the PI3K and PLC pathways by STX also promotes autophagic responses involved in removing Aβ and other neurotoxic proteins (as predicted by other studies; Uddin et al., 2018; Zhang Z. et al., 2021) remains to be explored. A schematic illustrating the potential mechanisms of STX-mediated neuroprotection is shown in Supplementary Figure 8C. In summary, our results indicate that the neuroprotective effects of STX involve complementary signaling pathways that in combination mitigate the effects of Aβ toxicity, supporting the hypothesis that STX might have therapeutic potential in patients at risk of AD.

## Data Availability

The raw data supporting the conclusions of this article will be made available by the authors, without undue reservation.

## Supplementary material

SUPPLEMENTARY FIGURE 1 Representative images of cultured cells used in different assays. **(A)** Examples of MC65 cell cultures used for experiments shown in Figure 1A. *Panel 1*: control cells maintained in Tet (Tet+). *Panel 2*: cell death at 72 hr caused by Tet removal (Tet-) to induce Aβ accumulation. *Panel 3*: STX protects cell viability in the absence of Tet (Tet-/STX+). *Panel 4*: the pan-PI3K inhibitor LY294002 (5 μM) blocked the protective effect of STX on viability (Tet-/STX+/LY). *Panel 5*: treatment with 5 μM LY294002 has no deleterious effect on MC65 cells in control conditions (Tet+/LY+). Scale = 20 μm. **(B)** Examples of mouse hippocampal neurons grown as sparse cultures above glial feeder layers, used for the experiments shown in Figure 3. *Panel 1*: Wt neuron grown in control medium. *Panel 2*: Wt neuron treated with STX. *Panel 3*: Wt neuron treated with 1.0 μM CAL101. *Panel 4*: Wt neuron treated with STX plus 1.0 μM CAL101. *Panel 5*: 5XFAD neuron grown in control medium. *Panel 6*: 5XFAD neuron treated with STX. *Panel 7*: 5XFAD neuron treated with 1.0 μM CAL101. Panel 8: 5XFAD neuron treated with STX plus 1.0 μM CAL101. Scale = 20 μm. **(C)** Examples of mouse hippocampal neurons grown as dense cultures, used for the experiments shown in Figure 5. *Panel 1*: Wt neurons at 3 days after plating. *Panel 2*: Wt neurons at 6 days after plating. *Panel 3*: Wt neurons at 10 days after plating. *Panel 4*: Wt neurons at 14 days after plating (day of treatment). Scale = 20 μm in panels 1-3; 5 μm in *panel 4*.

Supplementary FIGURE 2 Uncropped immunoblots used to analyze the protective effects of STX on Akt and Gsk3β phosphorylation levels in MC65 cells, quantified in Figure 2.

SUPPLEMENTARY FIGURE 3 Uncropped immunoblots used to analyze the effects of PI-103 on STX-dependent phosphorylation of Akt, Gsk3β, and ERK1/2, quantified in in Figure 5.

SUPPLEMENTARY FIGURE 4 Uncropped immunoblots used to analyze the effects of the p110δ-specific inhibitor CAL101 on STX-dependent phosphorylation of Akt, Gsk3β, and ERK1/2, quantified in Figure 6A.

SUPPLEMENTARY FIGURE 5 Uncropped immunoblots used to analyze the effects of the p110δβ-specific inhibitor TGX-221 on STX-dependent phosphorylation of Akt, Gsk3β, and ERK1/2, quantified in Figure 6B.

SUPPLEMENTARY FIGURE 6 Uncropped immunoblots used to analyze the effects of the p110δα-specific inhibitor HS-173 on STX-dependent phosphorylation of Akt, Gsk3β, and ERK1/2, quantified in Figure 6C.

SUPPLEMENTARY FIGURE 7 Uncropped immunoblots used to analyze the effects of the p110δγ-specific inhibitor IPI-549 on STX-dependent phosphorylation of Akt, Gsk3β, and ERK1/2, quantified in Figure 6D.

SUPPLEMENTARY FIGURE 8 **(A,B)** STX signaling may protect against the loss of neuronal Ca^2+^ homeostasis caused by Aβ. In cultured Wt hippocampal neurons, treatment with neurotoxic Aβ oligomers (gray bars) induced a significant increase in the relative intensity of nuclear CaN **(A)** and NFatC4 **(B)**, indirect markers of neuronal Ca^2+^ overload. Treatment with STX mitigated the effects of Aβ (yellow bars). Histograms show means +/− SEM. Statistics: one-way ANOVA with Tukey’s *post-hoc* comparisons; in **A**, alpha= 0.05. *N* = 12, F = 7.455, R^2^ = 0.3112. In **B**: *N* = 12, F = 12.31, R^2^ = 0.4273. **p*<0.05; ***p*<0.01; ****p*<0.001; *****p*<0.0001; ns = not significant. **(C)** Schematic illustration of two complementary pathways by which STX might protect neurons against the misregulation of Ca^2+^ homeostasis and mitochondrial function caused by Aβ. *Pathway #1*: Engagement of GqMER by STX induces PI3K-dependent phosphorylation of Akt that in turn phosphorylates and inactivates GSK3β. In hippocampal neurons, this response is predominantly mediated by the p110δ catalytic subunit of PI3K, with a more modest contribution by the p110β isoform. In this manner, STX can prevent the activation of downstream targets regulated by activated GSK3β, including chronic opening of the mitochondrial Permeability Transition Pore (mPTP) that results in the loss of ATP production, Ca^2+^ dyshomeostasis, cytochrome C (cyt C) release, induction of caspase 3 (casp3) activation, and engagement of cell death pathways (including apoptotic and necroptotic responses). Other GSK3β targets that might be modulated by STX include Bax (and other Bcl-2 family pro-apoptotic proteins) and tau, which can be hyperphosphorylated by sustained GSK3β activity and exacerbates the neurotoxic effects of Aβ. *Pathway #2*: STX also promotes Gαq-dependent activation of PLC, which in turn induces the sequential activation of PKCδ and PKA. PKCδ can also phosphorylate and inhibit GSK3β (complementing the beneficial effects of PI3K/Akt activity), while PKA activity can support normal Ca^2+^ responses via both AMPA and NMDA channels, which are dysregulated by neurotoxic Aβ and contribute to neurodegenerative responses.
