# Supplementary figures and images for: Neuroprotection against beta-amyloid toxicity by the novel estrogen receptor modulator STX requires convergent signaling pathways
Source: Front Mol Neurosci. 2025 Sep 12;18:1670646. doi: 10.3389/fnmol.2025.1670646 (PMC12464056; doi:10.3389/fnmol.2025.1670646)

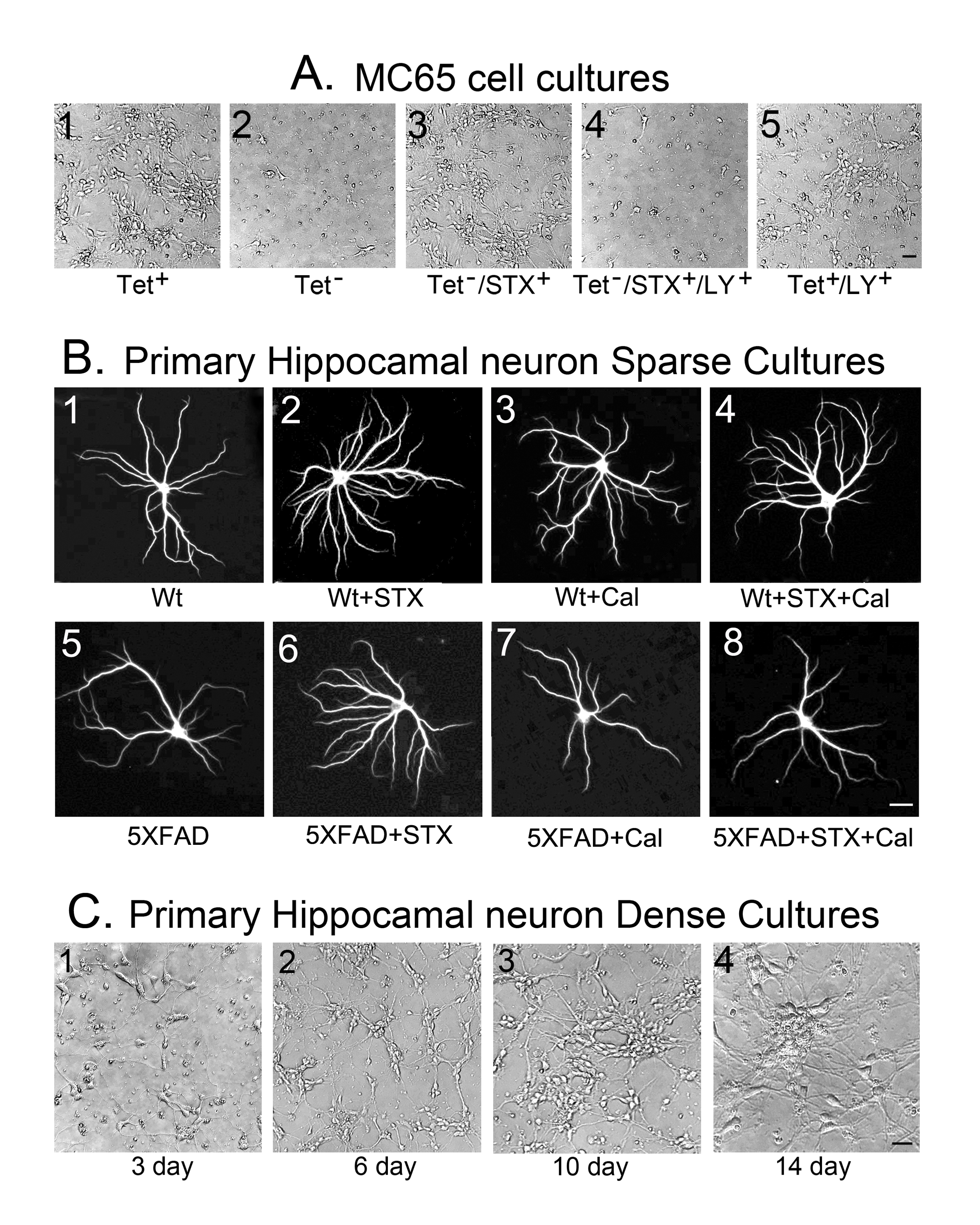

Supplement: SUPPLEMENTARY FIGURE 1 — Representative images of cultured cells used in different assays. (A) Examples of MC65 cell cultures used for experiments shown in Figure 1A. Panel 1: control cells maintained in Tet (Tet+). Panel 2: cell death at 72 hr caused by Tet removal (Tet-) to induce Aβ accumulation. Panel 3: STX protects cell viability in the absence of Tet (Tet-/STX+). Panel 4: the pan-PI3K inhibitor LY294002 (5 μM) blocked the protective effect of STX on viability (Tet-/STX+/LY). Panel 5: treatment with 5 μM LY294002 has no deleterious effect on MC65 cells in control conditions (Tet+/LY+). Scale = 20 μm. (B) Examples of mouse hippocampal neurons grown as sparse cultures above glial feeder layers, used for the experiments shown in Figure 3. Panel 1: Wt neuron grown in control medium. Panel 2: Wt neuron treated with STX. Panel 3: Wt neuron treated with 1.0 μM CAL101. Panel 4: Wt neuron treated with STX plus 1.0 μM CAL101. Panel 5: 5XFAD neuron grown in control medium. Panel 6: 5XFAD neuron treated with STX. Panel 7: 5XFAD neuron treated with 1.0 μM CAL101. Panel 8: 5XFAD neuron treated with STX plus 1.0 μM CAL101. Scale = 20 μm. (C) Examples of mouse hippocampal neurons grown as dense cultures, used for the experiments shown in Figure 5. Panel 1: Wt neurons at 3 days after plating. Panel 2: Wt neurons at 6 days after plating. Panel 3: Wt neurons at 10 days after plating. Panel 4: Wt neurons at 14 days after plating (day of treatment). Scale = 20 μm in panels 1-3; 5 μm in panel 4. [file Image_1.TIF]

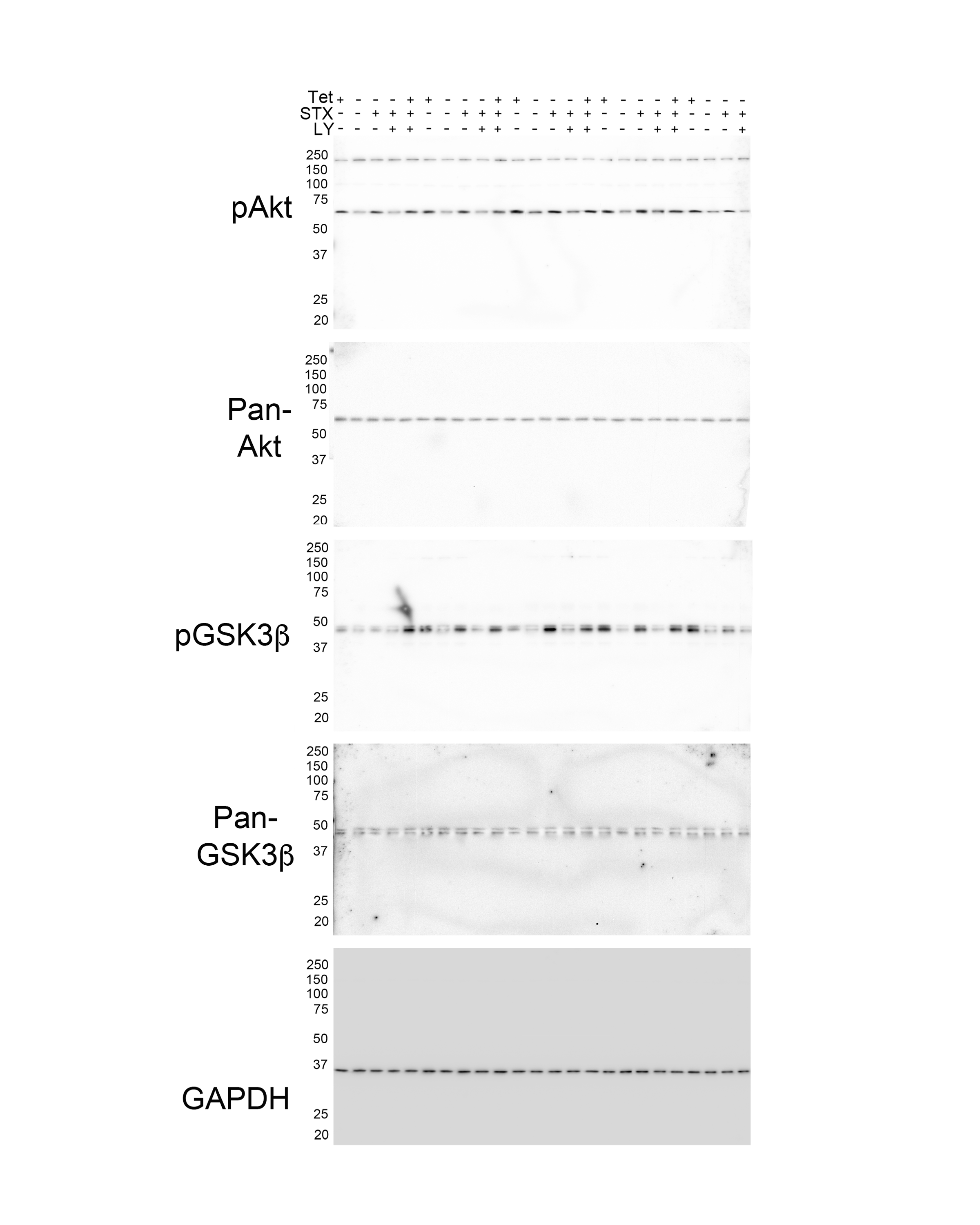

Supplement: Supplementary FIGURE 2 — Uncropped immunoblots used to analyze the protective effects of STX on Akt and Gsk3β phosphorylation levels in MC65 cells, quantified in Figure 2. [file Image_2.TIF]

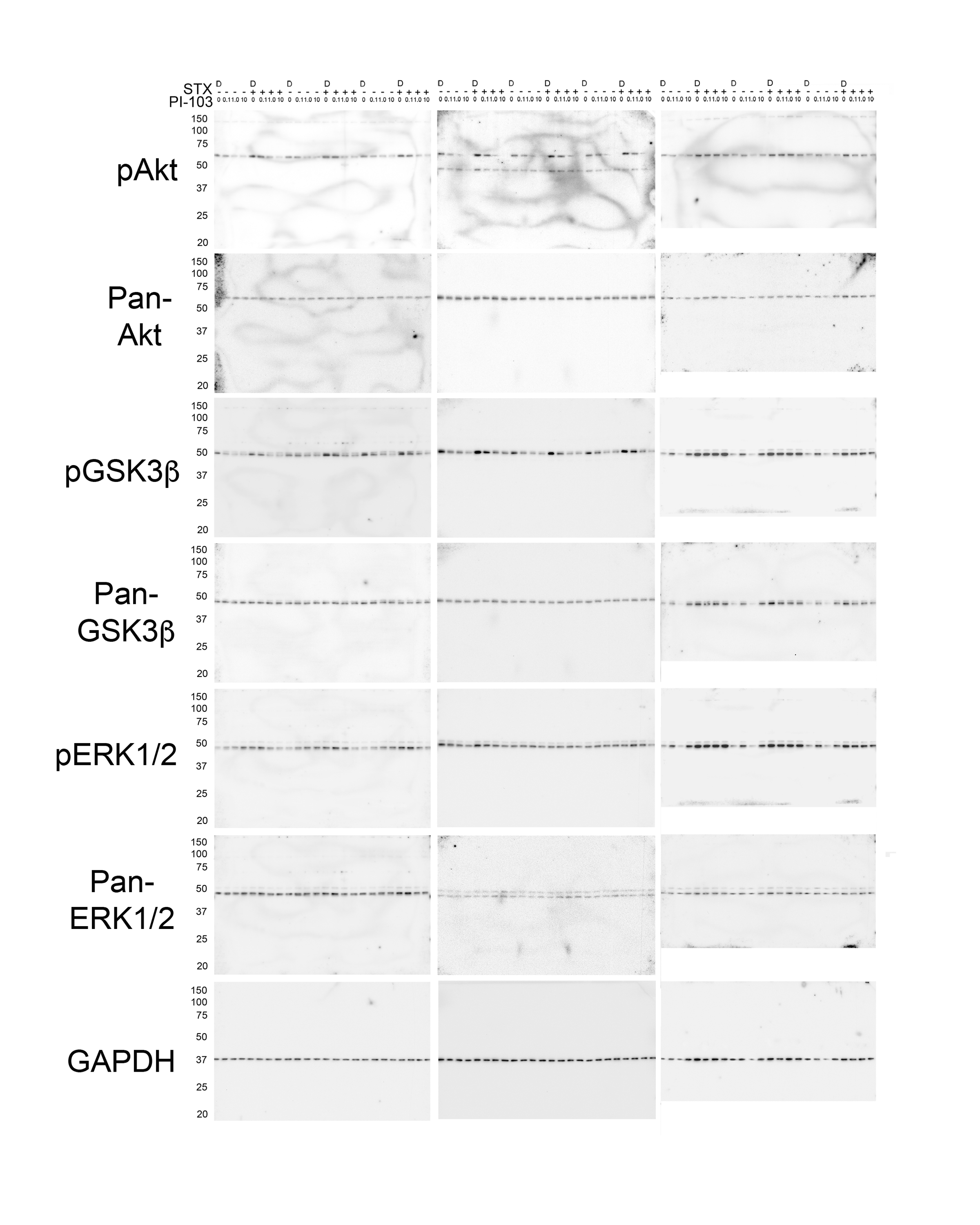

Supplement: SUPPLEMENTARY FIGURE 3 — Uncropped immunoblots used to analyze the effects of PI-103 on STX-dependent phosphorylation of Akt, Gsk3β, and ERK1/2, quantified in in Figure 5. [file Image_3.TIF]

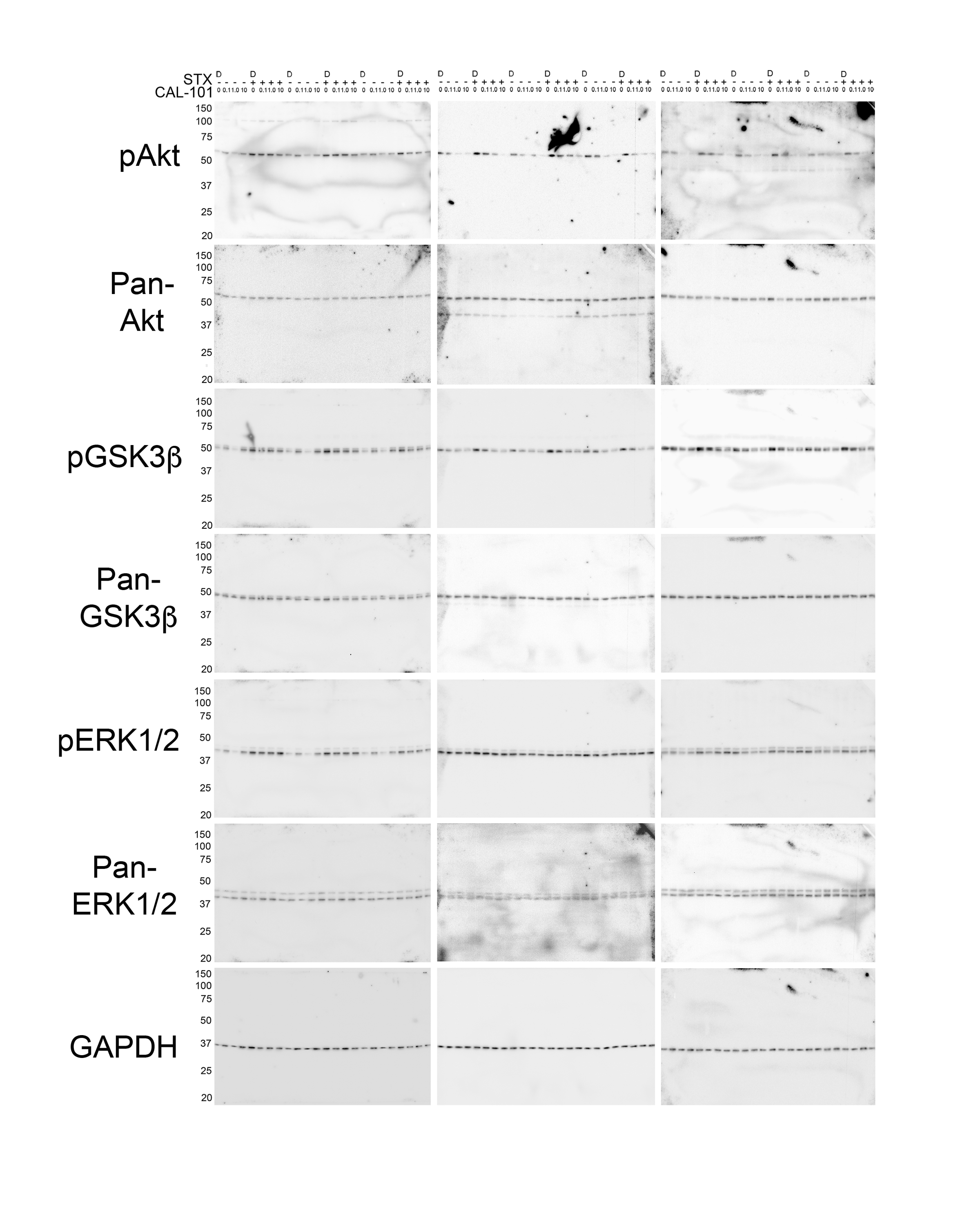

Supplement: SUPPLEMENTARY FIGURE 4 — Uncropped immunoblots used to analyze the effects of the p110δ-specific inhibitor CAL101 on STX-dependent phosphorylation of Akt, Gsk3β, and ERK1/2, quantified in Figure 6A. [file Image_4.TIF]

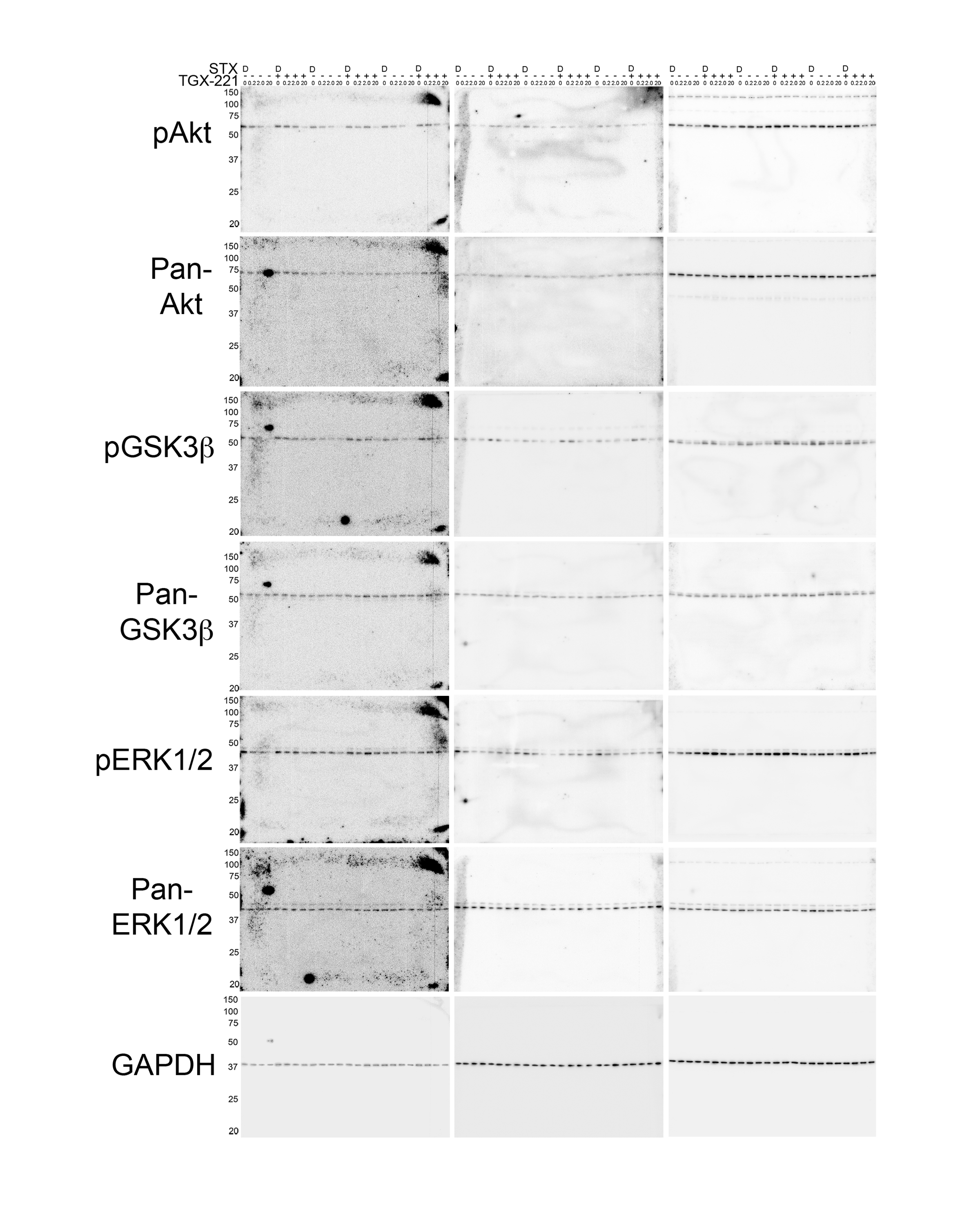

Supplement: SUPPLEMENTARY FIGURE 5 — Uncropped immunoblots used to analyze the effects of the p110δβ-specific inhibitor TGX-221 on STX-dependent phosphorylation of Akt, Gsk3β, and ERK1/2, quantified in Figure 6B. [file Image_5.TIF]

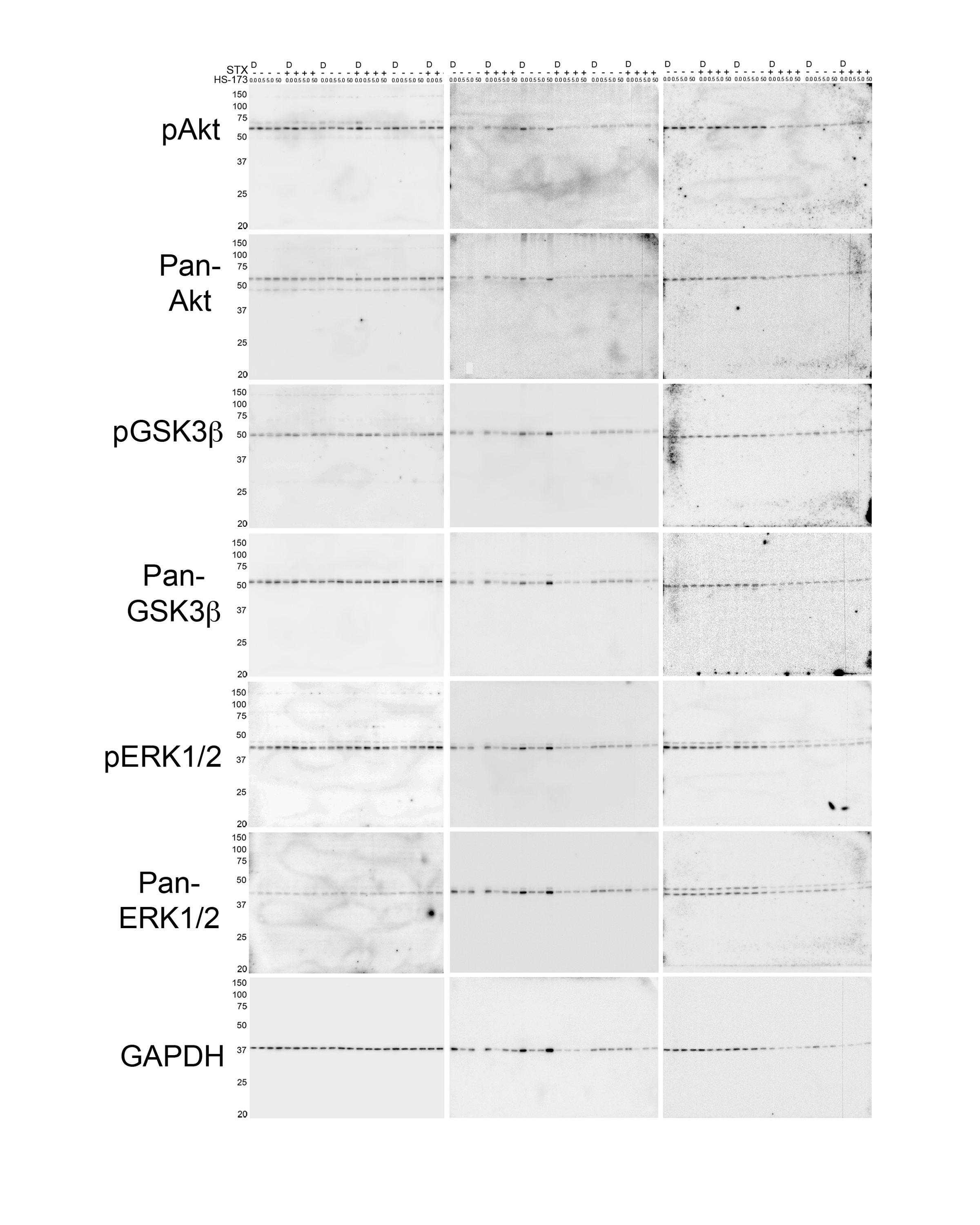

Supplement: SUPPLEMENTARY FIGURE 6 — Uncropped immunoblots used to analyze the effects of the p110δα-specific inhibitor HS-173 on STX-dependent phosphorylation of Akt, Gsk3β, and ERK1/2, quantified in Figure 6C. [file Image_6.TIF]

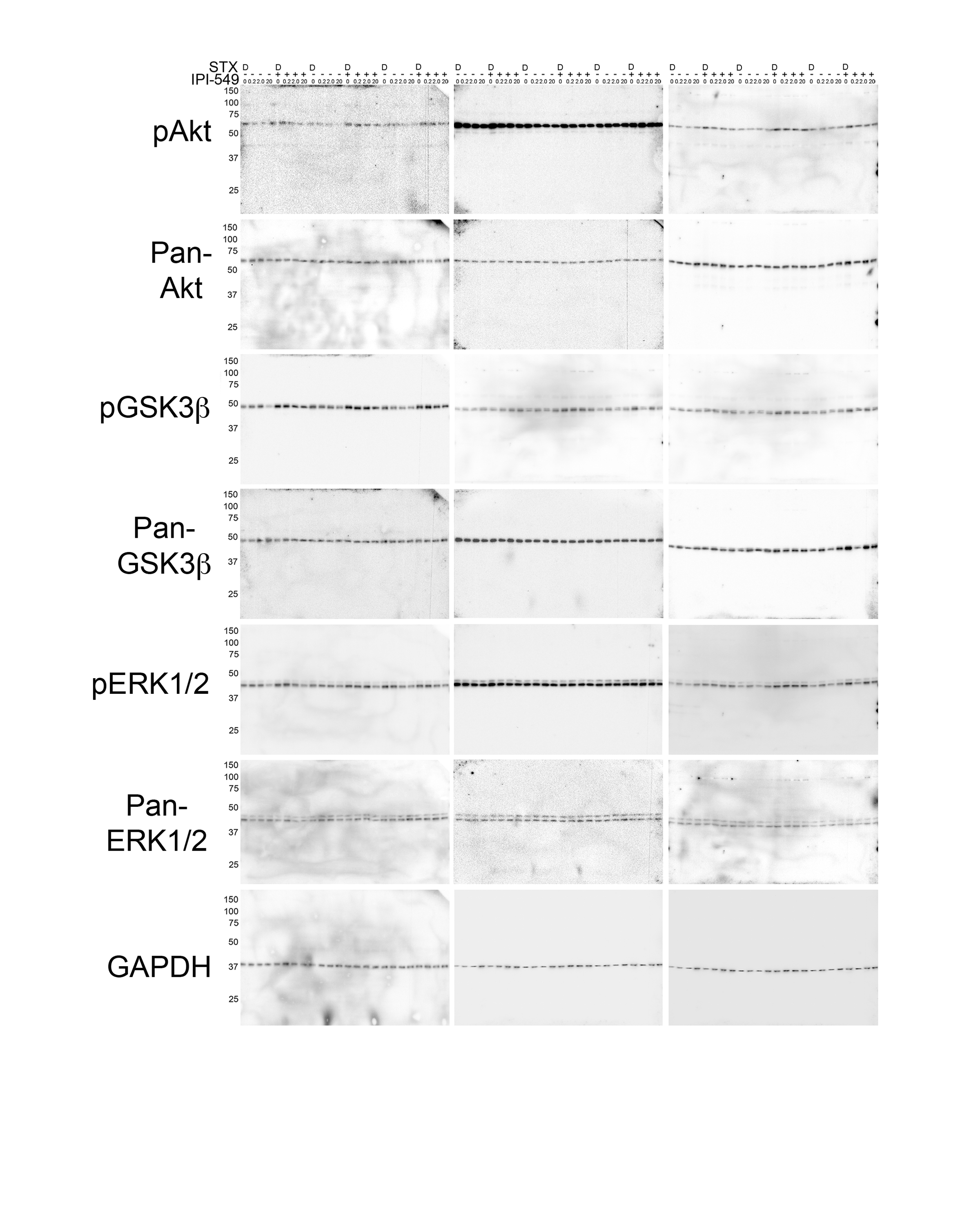

Supplement: SUPPLEMENTARY FIGURE 7 — Uncropped immunoblots used to analyze the effects of the p110δγ-specific inhibitor IPI-549 on STX-dependent phosphorylation of Akt, Gsk3β, and ERK1/2, quantified in Figure 6D. [file Image_7.TIF]

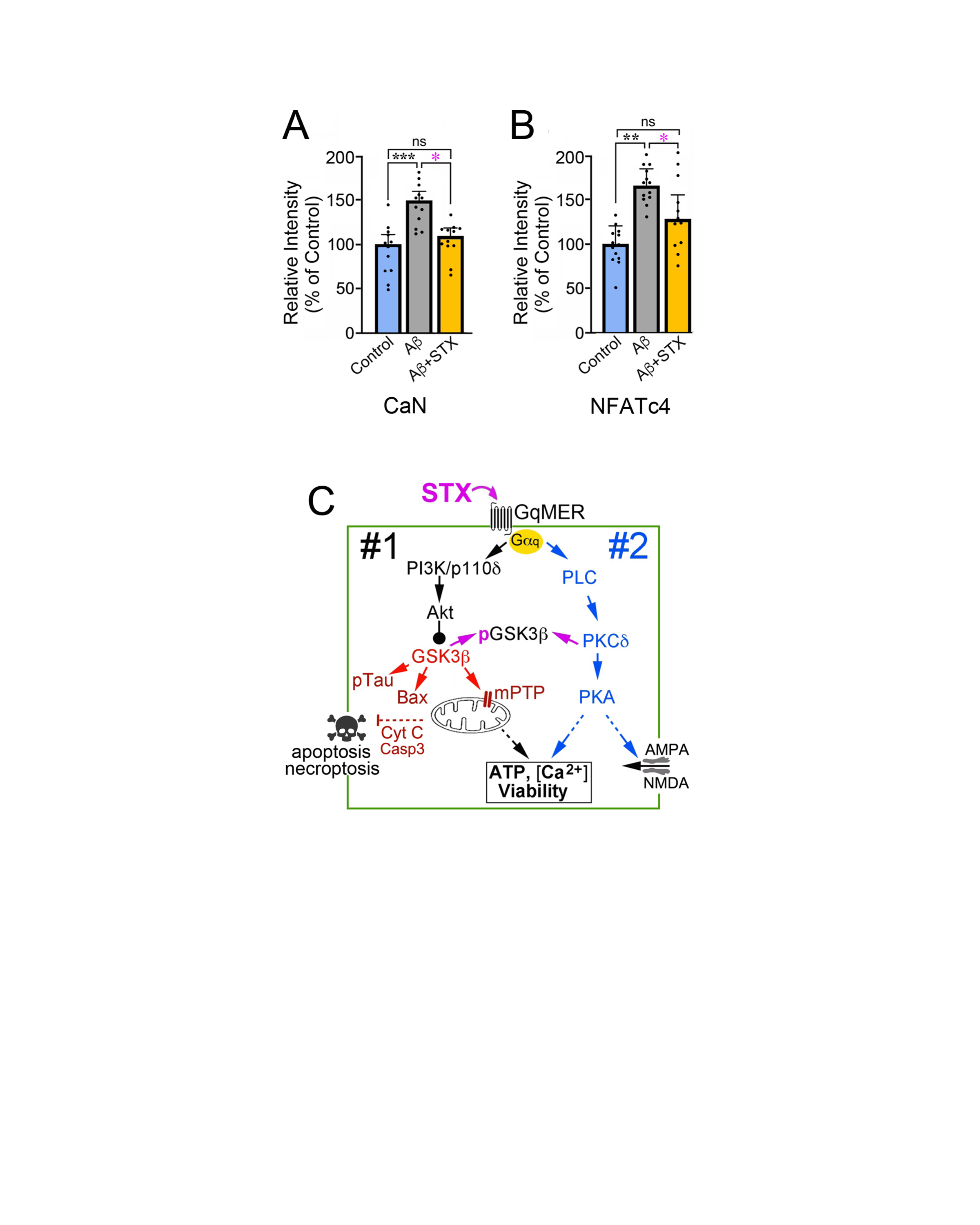

Supplement: SUPPLEMENTARY FIGURE 8 — (A,B) STX signaling may protect against the loss of neuronal Ca2+ homeostasis caused by Aβ. In cultured Wt hippocampal neurons, treatment with neurotoxic Aβ oligomers (gray bars) induced a significant increase in the relative intensity of nuclear CaN (A) and NFatC4 (B), indirect markers of neuronal Ca2+ overload. Treatment with STX mitigated the effects of Aβ (yellow bars). Histograms show means +/− SEM. Statistics: one-way ANOVA with Tukey’s post-hoc comparisons; in A, alpha= 0.05. N = 12, F = 7.455, R2 = 0.3112. In B: N = 12, F = 12.31, R2 = 0.4273. *p<0.05; **p<0.01; ***p<0.001; ****p<0.0001; ns = not significant. (C) Schematic illustration of two complementary pathways by which STX might protect neurons against the misregulation of Ca2+ homeostasis and mitochondrial function caused by Aβ. Pathway #1: Engagement of GqMER by STX induces PI3K-dependent phosphorylation of Akt that in turn phosphorylates and inactivates GSK3β. In hippocampal neurons, this response is predominantly mediated by the p110δ catalytic subunit of PI3K, with a more modest contribution by the p110β isoform. In this manner, STX can prevent the activation of downstream targets regulated by activated GSK3β, including chronic opening of the mitochondrial Permeability Transition Pore (mPTP) that results in the loss of ATP production, Ca2+ dyshomeostasis, cytochrome C (cyt C) release, induction of caspase 3 (casp3) activation, and engagement of cell death pathways (including apoptotic and necroptotic responses). Other GSK3β targets that might be modulated by STX include Bax (and other Bcl-2 family pro-apoptotic proteins) and tau, which can be hyperphosphorylated by sustained GSK3β activity and exacerbates the neurotoxic effects of Aβ. Pathway #2: STX also promotes Gαq-dependent activation of PLC, which in turn induces the sequential activation of PKCδ and PKA. PKCδ can also phosphorylate and inhibit GSK3β (complementing the beneficial effects of PI3K/Akt activity), while PKA activity can support no [file Image_8.TIF]
